# Epidemiology of invasive meningococcal disease worldwide from 2010–2019: a literature review

**DOI:** 10.1017/S0950268823000328

**Published:** 2023-03-06

**Authors:** Carmen Pardo de Santayana, Myint Tin Tin Htar, Jamie Findlow, Paul Balmer

**Affiliations:** 1 Pfizer Vaccine Medical Development, Scientific and Clinical Affairs, Paris, France; 2 Vaccine Medical Development and Scientific/Clinical Affairs, Pfizer Ltd, Tadworth, UK; 3 Pfizer Vaccine Medical Development and Scientific/Clinical Affairs, Collegeville, PA, USA

**Keywords:** Epidemiology, incidence, meningococcal disease, *Neisseria meningitidis*, vaccine-preventable diseases

## Abstract

The epidemiology of invasive meningococcal disease (IMD) is unpredictable, varies by region and age group and continuously evolves. This review aimed to describe trends in the incidence of IMD and serogroup distribution by age group and global region over time. Data were extracted from 90 subnational, national and multinational grey literature surveillance reports and 22 published articles related to the burden of IMD from 2010 to 2019 in 77 countries. The global incidence of IMD was generally low, with substantial variability between regions in circulating disease-causing serogroups. The highest incidence was usually observed in infants, generally followed by young children and adolescents/young adults, as well as older adults in some countries. Globally, serogroup B was a predominant cause of IMD in most countries. Additionally, there was a notable increase in the number of IMD cases caused by serogroups W and Y from 2010 to 2019 in several regions, highlighting the unpredictable and dynamic nature of the disease. Overall, serogroups A, B, C, W and Y were responsible for the vast majority of IMD cases, despite the availability of vaccines to prevent disease due to these serogroups.

## Introduction

Invasive meningococcal disease (IMD) is caused by *Neisseria meningitidis*, a Gram-negative diplococcus human pathogen that colonises the upper respiratory tract [1]. IMD most commonly manifests as meningitis, septicaemia or a combination of these; less common presentations include pneumonia, septic arthritis and pericarditis [2, 3]. Although relatively rare, IMD is a severe infectious disease. Initial symptoms are often nonspecific, and the disease may progress quickly to become fatal within hours [4]. IMD is associated with a 10% case fatality rate and lifelong, disabling sequelae affect 10%‒20% of survivors [5].

*N. meningitidis* can express a polysaccharide capsule, which is a primary factor determining its virulence [3, 6]. Twelve meningococcal serogroups have been identified: A, B, C, E, H, I, K, L, W, X, Y and Z [7]; however, 5 of the 12 serogroups (A, B, C, W and Y) have historically caused the majority of IMD and are associated with dynamic and unpredictable epidemiology that varies across time and regions [6, 8]. Recently, serogroup X has been associated with localised outbreaks in Africa [7]. IMD incidence also varies with age; although all ages are affected, incidence is highest in infants, followed by young children [9, 10]. Secondary peaks in incidence vary by region and have been observed in adolescents, young adults and older adults [9, 10].

Because of the unpredictable nature of IMD, continuous surveillance is necessary to monitor disease epidemiology in each country and region [11, 12]. Robust surveillance programs are essential for rapid detection of outbreak events and monitoring trends that define the disease burden over time [11]. Additionally, surveillance is necessary to guide public health strategies for preventing IMD [13]. Immunisation programs play an important role in disease prevention, and effective vaccines are available for preventing the major disease-causing serogroups A, B, C, W and Y [7]. Herein, we review global epidemiology data to describe trends in IMD incidence and serogroup distribution by age group and geography over time.

## Methods

The search strategy consisted of a literature review of PubMed publications and surveillance system data (grey literature) reporting IMD data. Additional detail regarding the searches, data review, inclusion/exclusion criteria, data extraction and collection, and data analysis is provided in the Supplementary Material.

## Results

**Fig. 1.**
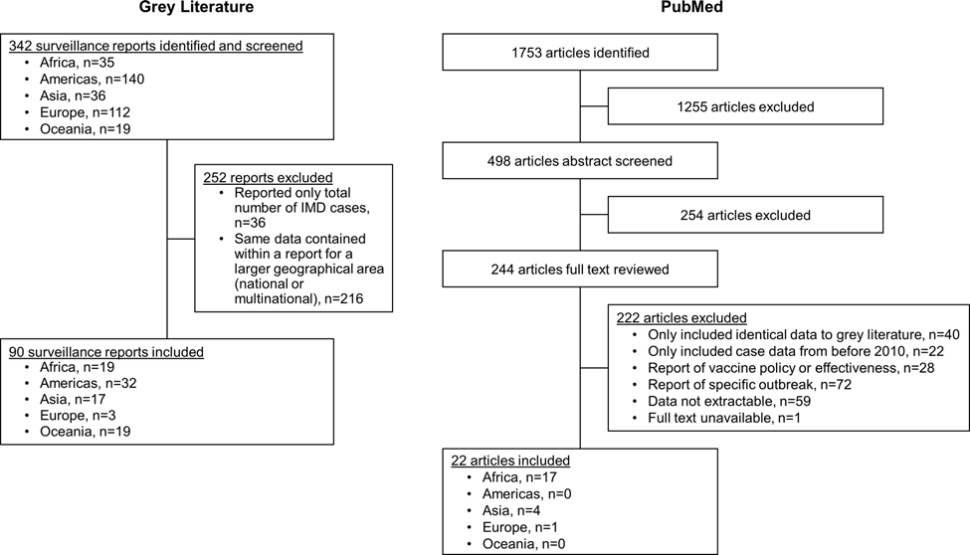
Flow chart of included grey literature reports and publications. IMD, invasive meningococcal disease.

A total of 90 grey literature reports and 22 publications were included out of 342 reports and 1753 publications initially screened (Fig. 1). The final data set included information for 77 countries. Grey literature data came from subnational, national or multinational reports, the last of which included the Surveillance Atlas of Infectious Diseases published by the European Centre for Disease Prevention and Control (ECDC), Sistema de Redes de Vigilancia de los Agentes Responsables de Neumonias y Meningitis Bacterianas (SIREVA II) and World Health Organization (WHO) Meningitis Weekly Bulletins: Inter country Support Team – West Africa. Publicly available data varied substantially across countries and global regions. Data are reported across the decade and for the final 3 years analysed (2017‒2019) to summarise recent trends.

### Incidence

#### Overall IMD incidence

Overall IMD incidence (per 100 000) was available for 10 non-European countries and 31 European countries (30 countries included within ECDC data and Ukraine national data; Table 1). IMD incidence was generally low across all regions during 2010‒2019 and ranged from 0.0 to 10.2 [14–25]; the highest incidences were reported for Niger (7.71 in 2015) and Burkina Faso (10.2 in 2012) [14, 15]. Excluding these countries, the consistently highest incidence was observed in New Zealand, reaching 2.8 in 2019 [22]. The consistently lowest incidences (<0.2) were observed in Saudi Arabia, the United States and Bulgaria [18, 19, 21]. Some countries, including Niger and Burkina Faso, had defined peaks associated with outbreaks [14, 15].

**Table 1.** Incidence of IMD worldwide from 2010 to 2019 (All ages; all serogroups: per 100 000 individuals; countries with available data)

Geographical region	2010	2011	2012	2013	2014	2015	2016	2017	2018	2019
Africa										
Burkina Faso [14]	–	2.1	10.2	2.1	2.0	2.3	–	–	–	–
Niger [15]	5.98	2.59	0.16	0.06	0.15	7.71	2.07	5.21	2.09	–
South Africa [16]	0.80	0.64	0.44	0.44	0.36	0.28	0.23	0.24	0.22	–
Asia & Middle East										
Russia [17]	1.16	1.16	0.99	0.89	0.68	0.67	0.50	0.58	0.70	–
Saudi Arabia [18]	–	–	–	–	0.01	0.02	0.02	0.03	0.03	–
Europe [19]	0.74	0.76	0.69	0.68	0.54	0.61	0.63	0.62	0.62	–
Austria	1.02	0.59	0.67	0.66	0.41	0.30	0.43	0.23	0.34	–
Belgium	0.85	1.02	1.11	1.20	0.78	0.88	0.95	0.85	1.02	–
Bulgaria	0.11	0.18	0.11	0.16	0.18	0.12	0.13	0.10	0.07	–
Croatia	–	–	0.96	0.61	0.78	0.99	0.72	0.89	0.76	–
Cyprus	0.12	0.00	0.70	0.23	0.47	0.47	0.71	0.47	0.12	–
Czech Republic	0.57	0.60	0.56	0.56	0.40	0.46	0.41	0.63	0.53	–
Denmark	1.19	1.29	1.00	0.98	0.80	0.39	0.67	0.68	0.62	–
Estonia	0.15	0.53	0.45	0.45	0.23	0.30	0.23	0.30	0.61	–
Finland	0.64	0.63	0.61	0.37	0.39	0.40	0.35	0.29	0.29	–
France	0.79	0.87	0.84	0.88	0.63	0.70	0.77	0.82	0.66	–
Germany	0.47	0.46	0.44	0.43	0.34	0.35	0.40	0.35	0.35	–
Greece	0.49	0.47	0.53	0.54	0.55	0.50	0.48	0.39	0.32	–
Hungary	0.37	0.67	0.51	0.47	0.33	0.36	0.48	0.40	0.41	–
Iceland	0.63	0.63	0.31	0.31	0.31	1.22	0.00	0.89	0.00	–
Ireland	2.15	1.95	1.31	1.67	1.64	1.45	1.80	1.48	1.82	–
Italy	0.25	0.26	0.23	0.27	0.26	0.31	0.38	0.33	0.28	–
Latvia	0.24	0.10	0.20	0.30	0.35	0.45	0.20	0.36	0.26	–
Lithuania	1.53	1.38	1.76	2.56	1.80	1.88	2.35	2.39	1.10	–
Luxembourg	0.20	0.39	0.57	0.56	0.55	0.18	0.17	0.00	0.50	–
Malta	0.48	1.45	0.72	2.84	3.03	1.14	1.33	0.43	0.84	–
The Netherlands	0.86	0.64	0.66	0.64	0.49	0.53	0.90	1.16	1.20	–
Norway	0.80	0.75	0.48	0.53	0.35	0.37	0.46	0.34	0.49	–
Poland	0.60	0.74	0.63	0.66	0.49	0.58	0.44	0.60	0.52	–
Portugal	0.75	0.73	0.65	0.58	0.50	0.63	0.37	0.48	0.55	–
Romania	0.26	0.34	0.35	0.26	0.34	0.25	0.28	0.25	0.33	–
Slovakia	0.69	0.39	0.57	0.33	0.42	0.44	0.42	0.68	0.66	–
Slovenia	0.44	0.63	0.44	0.53	0.39	0.78	0.34	0.44	0.87	–
Spain	0.87	0.92	0.72	0.56	0.31	0.45	0.56	0.58	0.84	–
Sweden	0.72	0.72	1.09	0.77	0.50	0.53	0.63	0.49	0.55	–
Ukraine [25]	–	–	–	–	–	–	0.63	0.77	0.64	0.71
United Kingdom	1.61	1.64	1.36	1.33	1.17	1.44	1.31	1.17	1.17	–
North America										
Canada [20]	0.45	0.50	0.43	0.34	0.28	0.29	0.26	0.32	–	–
United States [21]	–	–	–	–	–	0.12	0.12	0.11	0.10	–
Oceania										
New Zealand [22]	2.4	2.7	1.9	1.5	1.0	1.4	1.6	2.3	2.5	2.8
South America										
Brazil [23]	1.54	1.44	1.28	1.05	0.80	0.64	0.54	0.55	0.53	–
Chile [24]	–	–	0.67	0.70	0.78	0.67	0.58	0.44	0.41	0.36

IMD, invasive meningococcal disease.

A dash indicates no incidence data were available.

Varying trends in incidence were observed during 2010‒2018 [14–25]. Decreasing trends in incidence were observed in the United States, South Africa and Brazil [16, 21, 23]. Overall IMD incidence in Europe decreased slightly from 0.74 in 2010 to 0.62 in 2018 [19]. In line with this trend, decreasing incidence was observed in Denmark, Finland, Austria and the United Kingdom; however, increasing incidence was observed in Belgium and the Netherlands [19]. Some European countries, such as Spain, showed an initial decline followed by a more recent increase [19]. Globally, trends in incidence also fluctuated in Chile, New Zealand and Russia [17, 22, 24].

#### IMD incidence by age group

In countries and regions with available data, IMD occurred across all reported age groups (Supplementary Tables S1 and S2) [19–23]. The highest incidences were observed in infants (age <1 year), with rates often approximately 2- to 5-fold in this age group compared with young children (age 1‒4 years) and 10-fold compared with older age groups [19–23]. Particularly high incidence rates in infants during 2010‒2018 were observed in countries including New Zealand, where rates ranged from 10.2 to 47.7 per 100 000 and Ireland, which had rates of 19.3 to 38.8 (Supplementary Tables S1 and S2) [19, 22].

The second-highest incidences occurred among young children (age 1‒4 years) [19–23]. Lithuania had among the highest incidence rates in this age group, ranging from 6.8 to 18.2 during 2010‒2018 (Supplementary Table S2) [19].

A secondary peak in incidence among adolescents/young adults was observed in the United States, where annual IMD incidence was 0.1‒0.21 among those 16‒23 years of age *vs.* ≤0.1 among those 11‒15 or 24‒44 years of age in 2015‒2018 [21]. Similar secondary peaks in this age group were also observed in Canada, New Zealand and Europe overall but not Brazil (Supplementary Tables S1 and S2) [19, 20, 22, 23]. Secondary peaks in this age group varied by country and year within Europe; for example, the 15‒24-year age group in Sweden had consistently higher IMD incidence (0.58‒1.69) than those 5‒14 (0‒0.59) or 25‒49 (0.12‒0.39) years of age throughout 2010‒2018, whereas incidence among those 15‒24 years of age in Italy (0.30‒0.54) was similar to or lower than for those 5‒14 years of age (0.36‒0.54) during 2010‒2014 [19].

Increased incidence in older adults was observed in the United States (0.13‒0.15 among those ≥65 years of age during 2015‒2018), Canada (0.22‒0.41 among those ≥60 years of age during 2013‒2017) and New Zealand (1.3‒2.3 among those ≥70 years of age during 2015‒2018) but not Brazil (Supplementary Tables S1 and S2) [20–23]. In Europe, incidence among adults ≥50 years of age (0.30‒0.49) was higher than among adults 25‒49 years of age (0.19‒0.26) throughout 2010‒2018, with incidence particularly elevated among those ≥65 years of age (0.33‒0.63) [19]. This trend was echoed in many individual countries such as the United Kingdom, while other countries, such as the Czech Republic, did not exhibit this pattern (Supplementary Table S2) [19].

### Meningococcal serogroup distribution

#### Overall serogroup distribution of IMD

**Fig. 2.**
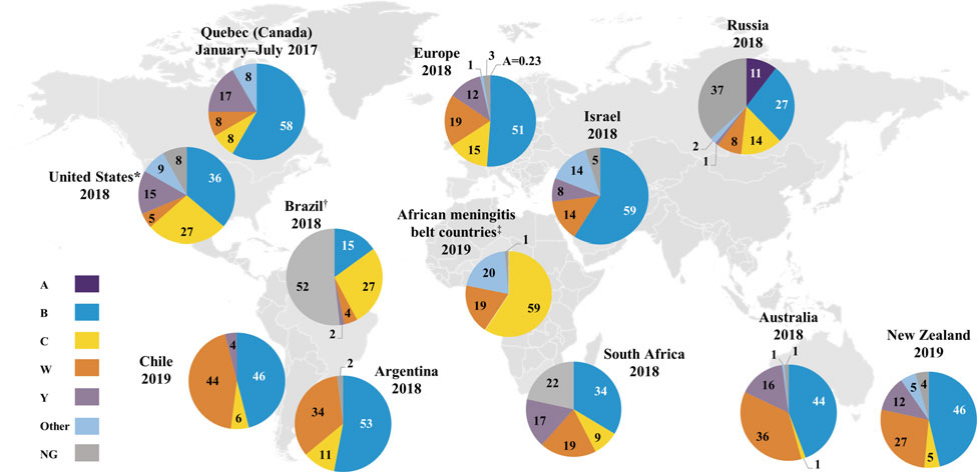
Percentage serogroup distribution of IMD cases worldwide from 2017 to 2019 (geographical regions with available data) [16, 17, 19, 21–24, 26, 27, 31–33]. Data from China, Colombia, Dominican Republic, Japan, Kuwait, Mozambique, Paraguay, South Korea, Uruguay and Venezuela are not shown. Percentages may not sum to 100% due to rounding. *Serogroup A is included in the “Other” category. ^†^Serogroups other than B, C, W and Y are included in the NG category. ^‡^Among the African meningitis belt countries (listed in the Supplementary Material), Benin, Burkina Faso, Cameroon, Central African Republic, Ghana, Mali, Niger, Nigeria, Senegal, South Sudan, Chad and Togo contributed serogroup data for 2019. IMD, invasive meningococcal disease; NG, nongroupable.

**Fig. 3.**
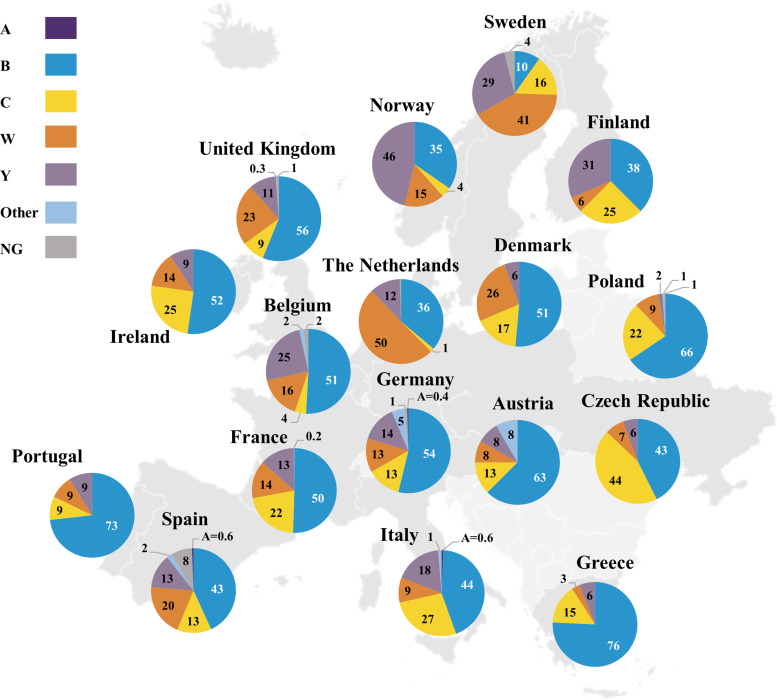
Percentage serogroup distribution of IMD cases in selected European countries in 2018 [19]. Percentages may not sum to 100% due to rounding. All serogroup A percentages are directly labelled. IMD, invasive meningococcal disease; NG, nongroupable.

Serogroup distribution data were available for Quebec (Canada), the United States, Argentina, Brazil, Chile, Colombia, Venezuela, Dominican Republic, Uruguay, Paraguay, African meningitis belt countries (listed in the Supplementary Material), Mozambique, South Africa, Israel, Kuwait, Belarus, Russia, China, Japan, South Korea, Australia, New Zealand and ECDC countries (Figs 2 and 3 and Supplementary Fig. S1) [16, 17, 19, 21–24, 26–37]. Globally, serogroups A, B, C, W and Y caused the majority of IMD, although the serogroup distribution varied across geographical regions at any given time and within the same geographical region over time.

Serogroup A was primarily observed in Burkina Faso, Chad, Niger and Russia (Supplementary Fig. S1a) but was not observed in Burkina Faso, Chad or Niger in recent years likely due to mass vaccination against serogroup A disease in this region [17, 33, 38]. Interestingly, serogroup A accounted for 10% of groupable IMD cases in China during 2015‒2017 and 14%‒25% of groupable IMD cases in Russia during 2016‒2018; however, 34%‒37% of cases in Russia had undetermined serogroups [17, 28]. Low serogroup A case numbers continue to occur in other countries, including 0.4%‒0.6% of cases in Germany, Italy and Spain in 2018 (Fig. 3) [19].

Serogroup B was the predominant disease-causing serogroup during the observation period in Europe, Israel, South Korea, Australia, New Zealand and Quebec (Supplementary Fig. S1a) [19, 22, 26, 27, 30, 31]. Although serogroup B predominated across Europe, serogroup B cases declined over time in many countries including the United Kingdom, France and Germany (Supplementary Fig. S1b) [19]. In other European countries including Poland and Italy, case numbers appeared stable during 2010‒2018 [19]. Serogroup B also predominated in South Africa, Russia and China in recent years (Supplementary Fig. S1a) [16, 17, 28]. Most recently (2017‒2019), serogroup B accounted for the highest percentage of IMD cases in almost all countries globally (Figs 2 and 3) [16, 17, 19, 21–24, 26, 27, 31–33]. Among the exceptions, serogroup B generally still caused a substantial proportion of IMD; these countries included Brazil (2018, 15%), Sweden (2018, 10%), Norway (2018, 35%), the Netherlands (2018, 36%) and the Czech Republic (2018, 43%) [19, 23]. Unlike other global regions, serogroup B was not responsible for any IMD in African meningitis belt countries in 2019 [33]. As noted, 37% of 2018 cases in Russia had unidentified serogroups; among those remaining, serogroup B constituted the largest percentage (27% of cases) [17].

During the observation period, serogroup C accounted for 66%, 41% and 55% of groupable IMD cases in Brazil, Colombia and Venezuela, respectively [32, 39]. Serogroup C was the second-highest disease-causing serogroup in African meningitis belt countries during 2010‒2019, accounting for 32% of cases [33, 37]. The presence of serogroup C in this region was generally transient; for example, a sharp increase in serogroup C cases was observed in Niger in 2015 due to an epidemic (Supplementary Fig. S1a) [38]. In Europe, serogroup C varied by country, with an overall increasing trend in the percentage of cases in the Czech Republic, Ireland, Italy and the United Kingdom and an overall decreasing trend in Austria, Belgium, Denmark, Germany, Norway, Poland and Sweden; no major trends were observed in other European countries (Supplementary Fig. S1b) [19]. An increase in the percentage of IMD due to serogroup C was observed in the United States, from 15% in 2015 to 27% in 2018; however, serogroup C case numbers increased from 54 in 2015 to 99 in 2016 and then decreased slightly to 90 cases in 2018 (Supplementary Fig. S1a) [21]. The most recent data (2017‒2019) show that serogroup C was present, albeit in varying percentages, across every global region (other than Israel in 2018, although Israel did record serogroup C cases in the preceding years; Figs 2 and 3, Supplementary Fig. S1) [16, 17, 19, 21–24, 26,27, 31–33]. Specifically, serogroup C accounted for 27% of cases in each of Brazil and the United States and a substantial percentage of IMD in eastern European countries (Poland, 22%; Czech Republic, 44%; Hungary, 34%; Slovakia, 17%) in 2018 [19, 21, 23].

Serogroup W was the predominant disease-causing serogroup in the African meningitis belt during 2010‒2019 (43% of IMD cases) [33, 37] and in Chile during 2012‒2018 (50%–73% of cases; Supplementary Fig. S1a) [24]. Outbreaks of serogroup W were observed in the African meningitis belt, with sharp increases in case numbers in Burkina Faso in 2012, Togo in 2016 and Chad in 2019 [33, 37]. Over the first half of the decade (2010‒2014), serogroup W predominated in Argentina (48%‒56% of cases) and South Africa (39%‒51% of serogrouped cases) [16, 32]. Over the second half of the decade, the percentage of serogroup W cases decreased over time in Argentina and South Africa, where serogroup B case numbers were stable or decreased but constituted an increasing percentage of IMD due to overall decreases in IMD, whereas increasing trends in the percentage of serogroup W cases were observed in Chad, Europe, Israel, Australia, New Zealand and Russia [16, 17, 19, 22, 27, 31–33, 37]. Since 2016, serogroup W case numbers have risen in Australia [31]. In New Zealand, increasing serogroup W case numbers led to serogroup W replacing serogroup C as the second-highest disease-causing serogroup, reaching 27% of IMD cases in 2019 [22]. Most recently (2017‒2019), the percentage of IMD cases due to serogroup W across countries in the southern hemisphere was generally substantial, ranging from 19% (South Africa, 2018) to 44% (Chile, 2019), with the exception of Brazil, where serogroup W did not expand to the same extent (2019, 4%; Fig. 2) [16, 22–24, 31, 32]. Similarly, 2018 data showed that serogroup W accounted for 3% (Greece) to 50% (the Netherlands) of cases across European countries, indicating varied expansion of serogroup W across the continent (Fig. 3) [19].

Serogroup Y, although not a predominant contributor to IMD globally, showed an increasing trend in case numbers from 2010 to 2018 in Europe, Israel, Australia and New Zealand (Supplementary Fig. S1a) [19, 22, 27, 31]. During 2013‒2014, serogroup Y accounted for 42% of IMD cases in Japan [29]. In other countries, serogroup Y comprised a stable proportion of cases during the observation period; this occurred both in countries with substantial presence of serogroup Y (e.g. the United States), and those where serogroup Y caused a relatively low percentage of IMD (e.g. Argentina; Supplementary Table S3) [21, 32]. Of note, in 2018, the percentage of IMD due to serogroup Y in northern European countries ranged from 29% in Sweden to 46% in Norway, where it was predominant (Fig. 3) [19].

Other serogroups, including X, Z and E, were minor contributors to IMD worldwide [16, 17, 19, 21–24, 26, 27, 30–33, 37]. Serogroup X cases were mostly limited to African meningitis belt countries, with the exception of sporadic cases.

#### IMD serogroup distribution by age group

Data on IMD serogroup distribution by age group were available from Argentina, Brazil, Chile, the United States, Australia, New Zealand and Europe [19, 21, 22, 24, 31, 32, 39]. Serogroups B, C, W and Y occurred across all age groups; however, trends were observed with respect to the predominant serogroup within age groups.

Serogroup B predominated across many age groups and regions during the study period [19, 21, 22, 24, 31, 32, 39]. Among infants and young children, serogroup B caused 40%‒100% of cases in Australia during 2010‒2018 and 41%‒80% of cases in the United States during 2015‒2018 (Supplementary Table S3) [21, 31]. In some countries, the percentage of cases caused by serogroup B was similarly high among adolescents/young adults as infants; this occurred in the United States (during 2015‒2018) and New Zealand (2018‒2019; Supplementary Table S3) [21, 22]. However, the dominance of serogroup B was frequently much more pronounced among younger compared with older age groups, with the relatively higher percentage of serogroup B disease in younger age groups persisting even in the context of broader decreases in serogroup B disease. For example, in Europe, serogroup B caused 75%‒84% of cases among age groups comprising infants and young children but 58%‒63% and 41%‒51% of cases among individuals 15‒24 and ≥50 years of age, respectively, during 2010‒2013, while percentages during 2016‒2018 were 63%‒70%, 48%‒50% and 29%‒30%, respectively [19]. Similarly, in Australia, percentages decreased from 86%‒100% and 44%‒88% among age groups comprising individuals ≤4 and ≥45 years of age, respectively, during 2010‒2012 to 40%‒62% and 10%‒37% during 2016‒2018 [31].

Serogroup C trends varied across age groups in Brazil, causing a stable majority (usually 50%‒80%) of IMD among those 15‒29 and ≥60 years of age during 2011‒2018 and decreasing percentages among infants and children 2‒4 years of age (Supplementary Table S3) [32, 39]. In other regions, serogroup C occurred in generally lower percentages across all age groups but was sometimes more frequent in adolescent/young adult and older age groups. For example, serogroup C caused 6%‒12% of IMD in Europe among those <1 and 1‒4 years of age during the study period, but 12%‒19% of cases among those 15‒24 and ≥50 years of age [19].

The expansion of serogroup W disease was evident in many age groups across countries (Supplementary Table S3) [19, 21, 22, 24, 31, 32, 39]. For example, serogroup W comprised ≤7% of cases across age groups in Australia in 2012 but rose to 17%‒50% by 2018 [31]. In many countries, percentages of cases caused by serogroup W rose more steeply in older adults compared with other age groups; this was the case for Europe overall as well as individual European countries including the Netherlands and the United Kingdom [19]. In the United States, serogroup W disease was generally more frequent among adults ≥45 years of age compared with younger age groups, although percentages in older age groups decreased during 2015‒2018 [21]. Unlike many other countries, decreasing trends in serogroup W case numbers were observed in Argentina and Chile among older adults and most other age groups during the study period [24, 32].

Across countries, serogroup Y often caused a higher percentage of IMD among older adults compared with other age groups (Supplementary Table S3) [19, 21, 22, 24, 31, 32]. This was true in New Zealand (during 2018‒2019) [22] and the United States (during 2015‒2018) [21], among other countries (Supplementary Table S3). Similarly, serogroup Y cases in Europe were much more frequent in adults ≥50 years of age (2010‒2018) followed by adolescents/young adults compared with younger age groups, with this trend holding true for many individual countries (Supplementary Table S3) [19].

## Discussion

This review evaluated 90 surveillance reports and 22 articles describing IMD epidemiology across 77 countries. IMD epidemiology varied with time and geographical region, and shifts in serogroup distribution were unpredictable. The highest IMD incidence rates were observed in African meningitis belt countries; however, relatively high incidence rates were also observed in other countries including New Zealand, Ireland, Lithuania and the United Kingdom. Local outbreaks, such as those in African meningitis belt countries, highlight the dynamic nature of IMD, which can be impacted by socioeconomic and sociocultural factors. For example, a recent study in northern Nigeria, which has experienced recurrent seasonal cerebrospinal meningitis epidemics, suggested factors such as multi-dwelling housing that resulted in overcrowding, inability to make informed decisions on family health, vaccine hesitancy and reluctance to take precautionary measures during outbreaks have made it difficult to control IMD in this region [40].

Global data showed that IMD affected all age groups, with the highest peaks in incidence observed in infants (age <1 year) followed by young children (age 1‒4 years). Secondary incidence peaks were observed in adolescents/young adults in the United States, Canada, New Zealand and many European countries, corresponding with increased carriage rates in these age groups [41]. Because meningococcal disease transmission occurs through social behaviours, behavioural changes within a given age group, such as increased travel, frequent attendance at bars and nightclubs and living in close quarters among adolescents/young adults, may influence relative IMD incidence rates across age groups [42, 43]. Increases in incidence were observed among older adults in the United States, Canada, New Zealand and many European countries.

Serogroup B accounted for the highest percentage of groupable IMD cases across most countries in the Americas, Europe, Australia and New Zealand. Across age groups, infants and young children were predominantly affected by serogroup B. In many countries, the percentage of serogroup B disease decreased over time as serogroup B cases declined and the number of cases due to other serogroups increased; however, this trend was not ubiquitous, as the percentages of serogroup B disease remained stable or even increased in some countries. Protein-based meningococcal serogroup B (MenB) vaccination programs have been implemented in a few countries [7], and reports of MenB vaccine effectiveness in the United Kingdom, Italy, Quebec and South Australia [44–47] suggest that MenB vaccination programs may influence serogroup epidemiology. Similarly, the number of serogroup C cases may have decreased over time as a result of increasing use of meningococcal serogroup C (MenC) vaccines in some countries [48]; however, serogroup C case numbers appeared to remain stable in several regions and still accounted for a substantial percentage of IMD in Brazil, the United States and some African and European countries.

The global expansion of serogroup W ST-11 complex (W:cc11) that occurred during the study period underscores the unpredictable and dynamic nature of IMD [7]. Serogroup W:cc11 predominated in Chile in 2012 and became the most or second most common disease-causing serogroup in several countries in South America, Europe, Australia and New Zealand [49]. In response to this increase, quadrivalent meningococcal serogroup A, C, W and Y (MenACWY) conjugate vaccines were introduced in several countries, and MenACWY vaccine impact on serogroup W disease epidemiology has been observed [7, 50, 51].

Serogroup Y disease was observed globally, with an increased number of cases noted in Europe, Israel, Australia and New Zealand, and contributed substantially to IMD in northern European countries, Australia and New Zealand [19, 22, 31]. The proportion of IMD caused by serogroup Y remained stable in other countries, including those with lower (e.g. Argentina) and higher (e.g. United States) serogroup Y proportions. Globally, older adults in several regions were particularly affected by serogroups W and Y.

As mentioned, the introduction of MenB and MenACWY conjugate vaccines into national immunisation programs, together with existing MenC conjugate vaccine programs, has likely influenced trends in IMD serogroup distribution [7, 44–47, 51]. Future vaccination programs may be designed to help prevent IMD in age groups with increased vulnerability to specific serogroups or IMD overall. In this vein, the strengthening of meningococcal surveillance programs will be fundamental to improving understanding of evolving IMD epidemiology. Relatedly, broad serogroup coverage through immunisation programs is also key to preventing IMD. Although IMD epidemiology is unpredictable, serogroups A, B, C, W and Y are likely to remain the major disease-causing serogroups. Despite the availability of vaccines to protect against these serogroups, existing immunisation programs do not cover all affected age groups [7]. To address these shortcomings, the WHO Global Road Map to Defeating Meningitis by 2030 seeks to reduce cases and deaths caused by vaccine-preventable disease by improving vaccine availability, immunisation coverage and disease surveillance [52].

We focused on IMD incidence and serogroup distribution, which are key parameters in basic epidemiology, and included recently available data (2017‒2019), thus expanding the current knowledge of disease trends. This review provides insight regarding geographical epidemiology of IMD in the decade preceding the coronavirus disease 2019 pandemic, which may be used for comparison in the future to assess the impact of pandemic mitigation measures on IMD epidemiology.

Our analysis was limited by availability of IMD data from grey literature sources; it was thus difficult to describe global trends. It is unknown to what extent the data presented here are representative of countries without published data. Additionally, differences among surveillance systems for different countries, such as heterogeneity of laboratory capacity, case definitions and reporting periods, limit the ability to make direct comparisons between countries and regions. Percentages of cases due to serogroups reported as ‘other,’ which typically included serogroups X, E and Z, or nongroupable isolates were not available for every country. Some countries reported a large number of ungrouped cases, thereby complicating determination of the true serogroup distribution. For example, 52% of 2018 cases in Brazil were reported as ‘ignored serogroups’; however, these cases likely represent predominantly serogroup B, C, W and Y cases that were not typed. In contrast, data from Europe published by the ECDC were almost fully typed. Increasing use of whole-genome sequencing will facilitate serogroup typing and allow for more accurate estimates of serogroup distributions. Another limitation is that, as noted, the data reviewed are from before the coronavirus disease 2019 pandemic and as such do not reflect considerable changes in meningococcal epidemiology associated with pandemic mitigation measures [53].

## Summary

IMD epidemiology is constantly evolving, and robust surveillance data are essential for its characterisation. Although global IMD incidence was generally low during 2010‒2019, relative predominance within circulating disease-causing serogroups varied substantially between countries. Changes in IMD epidemiology over time were likely influenced by natural fluctuations, emergence of virulent meningococcal clones (such as W:cc11), social trends and immunisation programs. Incidence was highest in infants, generally followed by young children; additional peaks were observed in adolescents/young adults and/or older adults in some countries. During the observation period, serogroup B generally predominated, while serogroup C remained ubiquitous but varied in proportion across global regions. Importantly, the number of IMD cases caused by serogroups W and Y notably increased during the study period. Although serogroup A no longer causes IMD in the African meningitis belt, serogroup A cases continue to occur in small numbers in other global regions. Thus, the vast majority of IMD cases were caused by serogroups A, B, C, W and Y, despite the availability of vaccines to prevent disease due to these serogroups.

## Data Availability

Data are available from the cited sources.

## Supplementary material

For supplementary material accompanying this paper visit http://doi.org/10.1017/S0950268823000328. click here to view supplementary material
