# Supplementary material for: Epidemiology of invasive meningococcal disease worldwide from 2010–2019: a literature review
Source: Epidemiol Infect. 2023 Mar 6;151:e57. doi: 10.1017/S0950268823000328 (PMC10126893; doi:10.1017/S0950268823000328)
Supplement: Supplementary file 1 [file S0950268823000328sup001.pdf]

Epidemiology and Infection

Epidemiology of Invasive Meningococcal Disease Worldwide From 2010–2019: A Literature Review

Carmen Pardo de Santayana, Myint Tin Tin Htar, Jamie Findlow, Paul Balmer

Supplementary Material

Search Strategy

Searches were restricted to publications and reports published between 1 January 2010 and 30 June 2020. The grey literature search was considered primary, and was undertaken on websites reporting subnational, national, and multinational IMD data that were previously known to the authors. Relevant IMD data within reports were identified by internal document searches of “meningococcal” and “meningitidis” in the corresponding language or review by native-speaking colleagues of the authors. Once identified, relevant report sections were translated to English using Google translation and Yandex® for text within figures and images.

The PubMed search terms were as follows: ((("Meningitidis"[Title/Abstract] OR "Meningo"[Title/Abstract] OR "Meningitides"[Title/Abstract] OR "neisseria meningitidis"[Title/Abstract]) NOT ("adaptive clinical trial"[Publication Type] OR "case rep orts"[Publication Type] OR "clinical study"[Publication Type] OR "clinical trial"[Publication Type] OR "clinical trial protocol"[Publication Type] OR "clinical trial, phase i"[Publication Type] OR "clinical trial, phase ii"[Publication Type] OR "clinical trial, phase iii"[Publication Type] OR "clinical trial, veterinary"[Publication Type] OR "controlled clinical trial"[Publication Type] OR "guideline"[Publication Type] OR "systematic review"[Publication Type] OR "randomized controlled trial"[Publication Type] OR "randomized controlled trial, veterinary"[Publication Type] OR "meta analysis"[Publication Type] OR "editorial"[Publication Type] OR "equivalence trial"[Publication Type] OR "observational study, veterinary"[Publication Type])) NOT ("econo"[Title/Abstract] OR "cost"[Title/Abstract] OR "cost effective"[Title/Abstract])) AND 2011/01/01:2020/06/15[Date - Publication] AND ("french"[Language] OR "italian"[Language] OR "english"[Language] OR "portuguese"[Language] OR "spanish"[Language]) AND ("human s"[All Fields] OR "humans"[MeSH Terms] OR "humans"[All Fields] OR "human"[All Fields]). Languages were restricted to English, Spanish, French, Italian, and Portuguese.

Data Review and Inclusion and Exclusion Criteria

Grey literature reports and publications were excluded if (1) they only contained IMD data for cases occurring before 2010, (2) they reported only the total number of IMD cases, or (3) data were not extractable. No inclusion or exclusion criteria were applied regarding diagnostic or confirmatory methods, or the case definition used.

Where multiple grey literature reports contained the same data for the same period, earlier reports and reports for the smaller geographical area(s) were excluded. For example, subnational reports were only used in the absence of national or multinational reports, and national reports were only used in the absence of multinational reports, with the exception of Brazil and Chile, where national reports were used when they provided the most accurate and complete data sets. Latin American countries reporting fewer than five IMD cases for any single year in the Sistema Regional de Vacunas II (SIREVA II) reports were also excluded.

Publications identified from the PubMed search were reviewed by two authors for application of inclusion and exclusion criteria. Publications reporting data from single-centred studies or specific outbreaks; meta-analyses; systematic reviews; clinical trials; case reports; models, simulations, or

extrapolations; guidelines; randomized trials; editorials; equivalence trials; nonhuman studies; health economic impact studies; vaccination policy studies; or vaccine effectiveness studies were excluded, as were publications that only contained data identical to data from grey literature reports. These criteria were applied stepwise in reviews of the title, abstract, and full text.

Data Extraction and Collection

The following data were extracted from grey literature reports and publications meeting the inclusion criteria: number of cases and/or percentage of IMD per serogroup for all ages, IMD (all serogroups) incidence (reported as N/10000) for all ages and per age group, number of cases and/or percentage of IMD per serogroup by age group, and the period and geographical area. Extracted data are consistent with those published at the date of initial download.

Analysis

Analyses of incidence and serogroup distribution of IMD were descriptive. For analysis and interpretation, subnational and national data were taken into account individually by country, with the exception of Europe and the African meningitis belt (24 countries, detailed below). Where only case numbers were available, serogroup distribution was calculated using Microsoft Excel.

African Meningitis Belt Countries [1]

Benin, Burkina Faso, Burundi*, Cameroon, Central African Republic, Chad, Côte d'Ivoire, Democratic Republic of Congo, Ethiopia, Gambia, Ghana, Guinea, Guinea Bissau*, Kenya*, Mali, Mauritania, Niger, Nigeria, Senegal, Sierra Leone*, South Sudan, Sudan, Tanzania*, Togo, Uganda*

*Did not report IMD serogroup data during 2010–2019

Supplementary Table S1. Incidence of IMD in Selected Countries by Age Group From 2010 to 2018 (Surveillance Data)

COUNTRY	AGE, Y	2010	2011	2012	2013	2014	2015	2016	2017	2018
**BRAZIL [2]**	<1	13.45	10.13	7.71	7.51	6.71	6.06	5.6	4.79	4.65
	1–4	5.18	4.47	3.18	2.43	1.83	2.11	1.21	1.46	1.27
	5–9	2.7	2.62	2.54	2.03	1.41	0.97	0.72	0.63	0.58
	10–14	2.1	1.81	1.87	1.31	1.19	0.79	0.71	0.67	0.57
	15–19	1.48	1.66	1.61	1.35	0.97	0.86	0.75	0.78	0.73
	20–29	0.88	0.92	0.85	0.72	0.55	0.4	0.39	0.45	0.5
	30–39	0.72	0.61	0.63	0.56	0.38	0.31	0.31	0.3	0.3
	40–49	0.65	0.72	0.77	0.72	0.45	0.35	0.33	0.38	0.37
	50–59	0.55	0.69	0.62	0.52	0.49	0.37	0.25	0.27	0.33
	≥60	0.41	0.58	0.47	0.34	0.27	0.24	0.3	0.27	0.28
**CANADA [3]**	<1	4.76	6.91	3.18	3.68	4.72	2.62	1.3	3.14	-
	1–4	1.47	2.17	1.1	1.17	0.91	0.91	0.83	0.51	-
	5–9	0.11	0.22	0.38	0.21	0.16	0.05	0.2	0.15	-
	10–14	0.26	0.36	0.69	0.16	0.11	0.21	0.1	0.15	-
	15–19	1.02	1.03	1.18	0.74	0.85	0.67	0.48	0.72	-
	20–24	0.65	0.47	0.5	0.25	0.17	0.33	0.25	0.79	-
	25–29	0.21	0.25	0.25	0.25	0.12	0.08	0.2	0.08	-
	30–39	0.15	0.13	0.11	0.11	0.06	0.14	0.1	0.1	-
	40–59	0.35	0.2	0.29	0.16	0.18	0.19	0.12	0.15	-
	≥60	0.29	0.49	0.28	0.4	0.22	0.31	0.4	0.41	-
**NEW ZEALAND [4,5]**	<1	47.7	38.5	19.8	18.4	10.2	22.0	18.6	23.1	28.2
	1–4	10.5	12.7	5.6	5.2	6.0	6.9	6.9	9.8	6.1
	5–9	2.4	3.5	1.4	-	-	-	1.6	1.5	2.8
	10–14	1.6	1.7	2.1	1.8	-	-	-	-	1.6
	15–19	3.7	4.7	4.8	3.9	-	2.2	3.1	5.4	6.0
	20–29	1.6	0.8	1.9	0.9	-	1.2	1.6	1.8	2.0
	30–39	-	1.2	1.3	-	-	-	-	-	1.3
	>40	0.7	1.0	0.7	0.7	-	-	-	-	-
	40–49	-	-	0.8	-	-	-	1.1	0.8	1.0
	50–59	-	-	1.1	-	-	-	-	2.3	1.0
	60–69	-	-	-	-	1.1	-	-	1.2	1.8
	≥70	-	-	-	-	-	1.3	1.3	2.3	2.2
**UNITED STATES [6]**	<1	-	-	-	-	-	1.14	0.93	0.63	0.83

1–4	-	-	-	-	-	0.2	0.21	0.13	0.18
5–10	-	-	-	-	-	0.04	0.05	0.07	0.04
11–15	-	-	-	-	-	0.04	0.04	0.04	0.03
16–23	-	-	-	-	-	0.15	0.21	0.2	0.1
24–44	-	-	-	-	-	0.1	0.09	0.07	0.07
45–64	-	-	-	-	-	0.1	0.08	0.08	0.09
≥65	-	-	-	-	-	0.13	0.13	0.15	0.14

IMD=invasive meningococcal disease.

A dash indicates no incidence data were available.

Supplementary Table S2. Incidence of IMD (Per 100000) in European Countries by Age Group From 2010 to 2014 (ECDC) [7]

	2010				2011				2012				2013				2014			
**COUNTRY**	**<1 y**	**1–4 y**	**15–24 y**	**≥50 y**	**<1 y**	**1–4 y**	**15–24 y**	**≥50 y**	**<1 y**	**1–4 y**	**15–24 y**	**≥50 y**	**<1 y**	**1–4 y**	**15–24 y**	**≥50 y**	**<1 y**	**1–4 y**	**15–24 y**	**≥50 y**
**AUSTRIA**	14.53	5.36	2.74	0.4	6.39	1.59	1.57	0.36	9.03	3.48	1.96	0.16	15.39	2.51	1.18	0.25	5.1	1.87	1.18	0.25
**BELGIUM**	9.46	3.19	1.83	0.25	12.29	4.66	1.73	0.65	14.76	5.56	2.02	0.39	11.75	6.29	2.24	0.53	11.98	3.44	1.28	0.38
**BULGARIA**	0	0	0	0	0	0	0	0	1.51	1.07	0.24	0	1.52	1.07	0.25	0.1	0	1.44	0	0.1
**CROATIA**	-	-	-	-	-	-	-	-	0	13.96	0.79	0	0	0	0.6	0	0	0	0.4	0
**CYPRUS**	0	0	0.76	0	0	0	0	0	10.27	0	2.24	0	0	0	0	0.37	21.66	0	0	0
**CZECH REPUBLIC**	6.68	2.89	1.31	0.11	10.07	2.76	1.27	0.21	11.03	3.73	0.91	0.16	12.88	2.54	0.85	0.21	5.62	2.39	0.44	0.08
**DENMARK**	6.33	6.85	2.95	0.7	12.55	5.35	2.45	0.89	15.18	5	0.85	1.08	8.58	3.14	2.37	0.48	12.46	2.02	0.83	0.86
**ESTONIA**	0	0	0	0	12.55	1.61	1.73	0.2	6.79	1.58	1.21	0.2	0	1.61	0.64	0.4	0	1.66	0.68	0
**EUROPE**	12.97	4.17	1.22	0.3	12.3	4.09	1.29	0.34	11.24	3.78	1.13	0.33	11.63	3.13	1.05	0.36	10.05	2.46	0.76	0.3
**FINLAND**	6.61	2.1	1.52	0.34	3.27	1.25	1.97	0.43	4.99	0.82	1.51	0.56	8.38	2.05	0	0.28	6.85	0.41	0.76	0.32
**FRANCE**	8.65	3.43	1.6	0.3	10.8	3.51	1.86	0.39	8.87	2.94	1.87	0.46	10.19	2.17	1.72	0.52	8.98	1.72	1.04	0.36
**GERMANY**	7.82	1.86	1.31	0.21	7.55	2.28	1.22	0.19	7.14	2.13	0.96	0.23	5.49	2.02	1.01	0.23	5.56	1.69	0.7	0.2
**GREECE**	6.96	4.33	0.97	0.1	5.43	2.24	1.07	0.12	2.83	3.58	1.77	0.09	8	2.7	0.95	0.19	9.57	2.79	1.14	0.16
**HUNGARY**	6.33	1.77	1.12	0.08	10.12	3.05	1.54	0.24	9.12	2.87	0.99	0.11	14.6	2.4	0.75	0.11	10.07	2.46	0.51	0.11
**ICELAND**	0	0	4.27	0	0	5.35	0	0	0	5.26	0	0	0	0	2.12	0	0	5.31	0	0
**IRELAND**	37.18	12.81	1.92	0.73	38.76	9.46	1.53	0.48	23.27	7.01	1.41	0.31	35.82	7.02	2.13	0.61	29.76	8.55	1.77	0.44
**ITALY**	4.65	1.2	0.34	0.1	3.28	1.03	0.54	0.11	3.01	1.13	0.37	0.12	3.24	1.08	0.42	0.18	4.13	1.08	0.3	0.18
**LATVIA**	0	3.34	0	0	5.2	1.13	0	0	0	0	0.38	0	10.15	1.23	0.41	0	0	2.54	0.44	0.13
**LITHUANIA**	40.82	18.17	0.65	0.27	9.81	6.83	4.14	0.44	29.81	10.94	3.12	0.35	59.09	14.93	2.24	1.22	36.83	10.72	1.54	0.35
**LUXEMBOURG**	0	0	1.68	0	17.08	0	1.64	0	0	8.43	0	0	16.63	4.12	0	0.58	0	12.02	0	0

**MALTA**	24.82	0	0	0.66	0	0	5.34	1.29	0	0	3.6	0.63	71.72	0	7.16	2.47	0	23.62	1.8	3.02
**THE NETHERLANDS**	13.54	4.73	1.43	0.46	8.15	3.52	1.18	0.35	10.58	4.33	0.88	0.29	8.54	3.26	0.63	0.5	5.85	2.2	0.68	0.36
**NORWAY**	1.61	2.48	3.35	0.43	4.87	2.43	1.09	0.66	4.96	0.4	1.98	0.24	4.96	1.58	0.75	0.35	3.37	1.19	0.3	0.46
**POLAND**	12.67	4.64	0.67	0.14	13.13	4.77	1.13	0.2	13.66	3.48	0.73	0.19	13.46	4.04	1.07	0.23	12.49	2.84	0.53	0.15
**PORTUGAL**	16.2	5.06	0.6	0.36	19.9	4.26	0.87	0.23	16.72	6.09	0.79	0.1	24.55	2.81	0.62	0.29	14.52	3.94	0.27	0.19
**ROMANIA**	5.07	1.05	0.24	0.15	4.08	2.81	0.32	0.15	7.56	2.59	0.45	0.07	6.14	1.58	0.21	0.04	7.2	1.92	0.18	0.15
**SLOVAKIA**	9.91	4.55	0.51	0.23	8.66	3.55	0.4	0.11	11.55	2.62	1.1	0.11	7.16	1.7	0.71	0.11	9.06	2.55	0.88	0
**SLOVENIA**	9.18	3.72	1.26	0	4.46	3.56	2.61	0.13	13.63	0	1.79	0.26	13.63	4.48	0	0	4.74	1.12	0.48	0.12
**SPAIN**	15.18	5.49	1.02	0.42	15.08	4.96	0.98	0.49	12.2	3.85	0.87	0.42	11.03	3.35	0.43	0.38	9.18	1.64	0.33	0.16
**SWEDEN**	0.89	1.83	1.61	0.75	4.31	1.79	1.52	0.66	0.89	1.76	1.69	1.55	1.76	0.87	0.97	1.23	3.51	0.43	0.58	0.75
**UNITED KINGDOM**	33.53	8.93	1.85	0.68	28	9.44	1.81	0.8	24.12	8.02	1.42	0.67	23.38	6.31	1.59	0.69	21.01	4.82	1.43	0.8

ECDC=European Centre for Disease Prevention and Control; IMD=invasive meningococcal disease.

A dash indicates no incidence data were available.

Supplementary Table S2 (continued). Incidence of IMD (Per 100000) in European Countries by Age Group From 2015 to 2018 (ECDC) [7]

	2015				2016				2017				2018			
**COUNTRY**	**<1 y**	**1–4 y**	**15–24 y**	**≥50 y**	**<1 y**	**1–4 y**	**15–24 y**	**≥50 y**	**<1 y**	**1–4 y**	**15–24 y**	**≥50 y**	**<1 y**	**1–4 y**	**15–24 y**	**≥50 y**
**AUSTRIA**	11.18	0.92	0.5	0.09	7.2	1.2	1.47	0.12	6.91	0.59	0.4	0.14	2.31	1.16	0.4	0.23
**BELGIUM**	15.22	2.69	1.51	0.63	10.62	3.3	1.66	0.56	10.68	2.55	1.67	0.57	5.02	5.96	1.84	0.75
**BULGARIA**	4.46	0.37	0.42	0.03	1.53	1.49	0.15	0.03	4.64	0.38	0.3	0.03	0	0.38	0.16	0
**CROATIA**	7.61	1.81	0.61	0.29	16.11	6.82	0.63	0	26.74	7.02	1.71	0.06	16.45	6.6	1.76	0
**CYPRUS**	10.83	0	0.84	0.72	0	10.47	0	0	0	0	1.71	0.35	0	0	0	0.34
**CZECH REPUBLIC**	12.73	0.89	1.01	0.15	5.42	2.05	1.24	0.08	10.66	2.94	1.38	0.3	9.63	2.02	0.81	0.22
**DENMARK**	7	0.83	0.82	0.28	3.41	1.69	1.22	0.83	4.84	1.27	1.35	0.81	4.86	3.35	0.68	0.53
**ESTONIA**	0	1.71	0	0	7.12	1.77	0	0	0	1.78	0.77	0	7.25	0	0	0.78
**EUROPE**	10.01	2.8	0.95	0.39	8.54	2.69	1.06	0.44	8.35	2.53	0.99	0.47	8.4	2.41	0.95	0.49
**FINLAND**	1.73	2.06	1.08	0.27	1.8	0	0.94	0.45	1.88	0	0.63	0.4	1.98	0.44	0.64	0.26
**FRANCE**	8.41	2.02	1.32	0.44	10.1	2.17	1.23	0.53	9.48	2.11	1.3	0.67	8.29	1.92	1.12	0.42
**GERMANY**	6	1.95	0.83	0.21	5.64	1.57	0.89	0.26	4.95	1.25	0.65	0.26	4.71	1.01	0.69	0.25
**GREECE**	6.54	1.96	0.9	0.23	4.37	2.58	1.28	0.18	8.52	1.57	0.73	0.18	4.47	2.89	0.37	0.13
**HUNGARY**	14	3.06	0.17	0.11	9.81	3.86	0.62	0.16	9.49	3.82	0.09	0.08	18.07	1.62	0.19	0.16
**ICELAND**	22.95	16.37	0	0	0	0	0	0	24.7	0	2.12	0.91	0	0	0	0
**IRELAND**	19.98	4.72	2.28	0.79	28.91	5.54	3.48	1.05	22.19	5.69	1.89	1.3	19.3	6.99	3.17	1.27
**ITALY**	4.03	0.83	0.69	0.2	4.59	1.05	0.88	0.19	3.21	0.93	0.58	0.23	2.63	0.86	0.51	0.23
**LATVIA**	18.46	1.27	0	0.25	4.57	0	0.5	0	13.74	1.17	0.53	0.13	4.84	0	0	0.38
**LITHUANIA**	19.76	14.15	2.38	0.17	41.32	13.33	3.07	0.95	36.17	16.63	3.28	0.6	6.95	10	1.94	0.34
**LUXEMBOURG**	0	0	0	0.54	0	0	0	0	0	0	0	0	0	0	4.23	0

**MALTA**	0	11.57	0	1.18	0	5.64	1.83	0	21.64	5.53	0	0	22.44	0	0	1.12
**THE NETHERLANDS**	6.87	1.4	1.01	0.51	8.22	2.26	1.49	0.96	9.29	3	2.14	1.22	8.85	3.58	1.7	1.48
**NORWAY**	0	1.2	0.89	0.28	5.06	0.81	0.45	0.72	0	0.41	0.75	0.49	0	2.06	0.9	0.43
**POLAND**	12.54	3.65	0.6	0.26	11.32	2.96	0.48	0.19	12.52	3.45	0.81	0.27	11.41	3.19	0.64	0.22
**PORTUGAL**	10.94	3.54	0.9	0.38	9.35	2.57	0.09	0.21	12.63	3.22	0.18	0.3	20.87	3.54	0.37	0.23
**ROMANIA**	3.13	1.44	0.23	0.18	5.51	0.93	0.32	0.15	5.05	1.68	0.33	0.06	5.51	1.26	0.19	0.22
**SLOVAKIA**	16.21	3.45	0.46	0.05	10.69	3.48	0.31	0.16	15.44	5.74	0.33	0.21	18.73	5.24	0.51	0.21
**SLOVENIA**	18.92	6.81	0.99	0.12	4.85	2.31	0	0	4.92	4.7	1.02	0.12	4.94	4.8	2.06	0.35
**SPAIN**	7.74	2.13	0.4	0.37	9.3	2.97	0.67	0.41	7.81	2.81	0.78	0.52	9.68	3.62	0.86	0.94
**SWEDEN**	0.86	1.07	0.75	0.82	1.73	1.06	1.61	0.72	1.68	0.84	1.03	0.48	2.57	0.62	0.95	0.89
**UNITED KINGDOM**	21.74	6.71	1.73	1.12	11.67	5.4	1.8	1.2	10.74	4.35	1.54	1.05	14.31	3.36	1.71	1.09

ECDC=European Centre for Disease Prevention and Control; IMD=invasive meningococcal disease.

A dash indicates no incidence data were available.

Supplementary Table S3. Serogroup Distribution of IMD Worldwide by Geography, Age Group, and Year*

Geographical Region	Age, y	Year	Serogroup B Cases		Serogroup C Cases		Serogroup W Cases		Serogroup Y Cases		Other/NG Cases	
			n	%	n	%	n	%	n	%	n	%
Argentina [8-10]	All ages	2010	56	41.8	7	5.2	66	49.3	4	3.0	1	0.7
		2011	66	43.4	5	3.3	73	48.0	7	4.6	1	0.7
		2012	66	38.2	4	2.3	96	55.5	6	3.5	1	0.6
		2013	65	41.9	9	5.8	77	49.7	3	1.9	1	0.6
		2014	70	47.0	2	1.3	73	49.0	4	2.7	0	0.0
		2015	48	51.6	8	8.6	34	36.6	2	2.2	1	1.1
		2016	44	55.0	5	6.3	26	32.5	5	6.3	0	0.0
		2017	45	59.2	8	10.5	18	23.7	5	6.6	0	0.0
		2018	34	53.1	7	10.9	22	34.4	0	0.0	1	1.6
	<1	2010	15	32.6	1	2.2	29	63.0	1	2.2	0	0.0
		2011	30	48.4	1	1.6	28	45.2	3	4.8	0	0.0
		2012	27	41.5	1	1.5	33	50.8	4	6.2	0	0.0
		2013	26	44.8	1	1.7	30	51.7	1	1.7	0	0.0
		2014	32	47.1	2	2.9	32	47.1	2	2.9	0	0.0
		2015	19	65.5	2	6.9	8	27.6	0	0.0	0	0.0
		2016	18	58.1	2	6.5	9	29.0	2	6.5	0	0.0
		2017	14	66.7	0	0.0	7	33.3	0	0.0	0	0.0
		2018	11	73.3	2	13.3	2	13.3	0	0.0	0	0.0
	1	2010	6	46.2	1	7.7	5	38.5	1	7.7	0	0.0
		2011	7	36.8	1	5.3	10	52.6	1	5.3	0	0.0
		2012	11	42.3	0	0.0	15	57.7	0	0.0	0	0.0
		2013	4	33.3	2	16.7	6	50.0	0	0.0	0	0.0
		2014	4	33.3	0	0.0	8	66.7	0	0.0	0	0.0
		2015	8	57.1	0	0.0	6	42.9	0	0.0	0	0.0
		2016	3	33.3	0	0.0	5	55.6	1	11.1	0	0.0
		2017	8	57.1	0	0.0	5	35.7	1	7.1	0	0.0
		2018	7	70.0	0	0.0	3	30.0	0	0.0	0	0.0
	2–4	2010	10	62.5	2	12.5	3	18.8	1	6.3	0	0.0
		2011	9	47.4	0	0.0	9	47.4	1	5.3	0	0.0
		2012	9	34.6	0	0.0	16	61.5	1	3.8	0	0.0
		2013	14	48.3	2	6.9	13	44.8	0	0.0	0	0.0
		2014	12	63.2	0	0.0	6	31.6	1	5.3	0	0.0
		2015	6	60.0	1	10.0	3	30.0	0	0.0	0	0.0
		2016	5	45.5	2	18.2	3	27.3	1	9.1	0	0.0
		2017	14	73.7	3	15.8	1	5.3	1	5.3	0	0.0
		2018	3	50.0	0	0.0	3	50.0	0	0.0	0	0.0
	15–29	2011	7	43.8	0	0.0	7	43.8	1	6.3	1	6.3
		2012	5	41.7	2	16.7	4	33.3	0	0.0	1	8.3
		2013	3	27.3	1	9.1	6	54.5	1	9.1	0	0.0
		2014	7	63.6	0	0.0	4	36.4	0	0.0	0	0.0
		2015	2	33.3	2	33.3	1	16.7	1	16.7	0	0.0
		2016	4	50.0	0	0.0	4	50.0	0	0.0	0	0.0
		2017	2	66.7	1	33.3	0	0.0	0	0.0	0	0.0
		2018	3	33.3	0	0.0	6	66.7	0	0.0	0	0.0
	≥60	2011	0	0.0	0	0.0	7	100.0	0	0.0	0	0.0
		2012	1	8.3	0	0.0	10	83.3	1	8.3	0	0.0
		2013	0	0.0	0	0.0	5	83.3	1	16.7	0	0.0
		2014	1	10.0	0	0.0	9	90.0	0	0.0	0	0.0
		2015	1	16.7	0	0.0	5	83.3	0	0.0	0	0.0
		2016	1	33.3	0	0.0	1	33.3	1	33.3	0	0.0
		2017	0	0.0	0	0.0	2	100.0	0	0.0	0	0.0
		2018	3	42.9	2	28.6	2	28.6	0	0.0	0	0.0
Australia† [11-13]	All ages	2010	167	78.0	16	7.5	9	4.2	7	3.3	15	7.0
		2011	179	74.3	9	3.7	11	4.6	15	6.2	27	11.2
		2012	161	77.4	11	5.3	7	3.4	15	7.2	14	6.7
		2013	104	72.7	8	5.6	12	8.4	15	10.5	4	2.8
		2014	129	78.2	3	1.8	16	9.7	13	7.9	4	2.4

Geographical Region	Age, y	Year	Serogroup B Cases		Serogroup C Cases		Serogroup W Cases		Serogroup Y Cases		Other/NG Cases	
			n	%	n	%	n	%	n	%	n	%
		2015	108	62.1	2	1.1	36	20.7	22	12.6	6	3.4
		2016	87	35.8	3	1.2	107	44.0	40	16.5	6	2.5
		2017	137	36.6	14	3.7	141	37.7	75	20.1	7	1.9
		2018	123	44.2	4	1.4	101	36.3	44	15.8	6	2.2
	<1	2010	25	92.6	2	7.4	-	-	-	-	-	-
		2011	32	100.0	0	0.0	-	-	-	-	-	-
		2012	24	88.9	1	3.7	0	0.0	1	3.7	1	3.7
		2013	21	91.3	0	0.0	2	8.7	0	0.0	0	0.0
		2014	24	82.8	1	3.4	2	6.9	1	3.4	1	3.4
		2015	9	56.3	1	6.3	2	12.5	0	0.0	4	25.0
		2016	14	58.3	0	0.0	8	33.3	2	8.3	0	0.0
		2017	16	40.0	0	0.0	17	42.5	6	15.0	1	2.5
		2018	23	62.2	0	0.0	10	27.0	2	5.4	2	5.4
	1–4	2010	36	100.0	0	0.0	-	-	-	-	-	-
		2011	30	100.0	0	0.0	-	-	-	-	-	-
		2012	30	85.7	0	0.0	2	5.7	0	0.0	3	8.6
		2013	23	95.8	0	0.0	0	0.0	0	0.0	1	4.2
		2014	16	94.1	1	5.9	0	0.0	0	0.0	0	0.0
		2015	20	83.3	0	0.0	2	8.3	1	4.2	1	4.2
		2016	11	42.3	0	0.0	11	42.3	1	3.8	3	11.5
		2017	21	45.7	0	0.0	21	45.7	1	2.2	3	6.5
		2018	22	59.5	0	0.0	14	37.8	1	2.7	0	0.0
	15–19	2010	29	96.7	1	3.3	-	-	-	-	-	-
		2011	36	100.0	0	0.0	-	-	-	-	-	-
		2012	22	78.6	1	3.6	2	7.1	1	3.6	2	7.1
		2013	22	84.6	1	3.8	3	11.5	0	0.0	0	0.0
		2014	22	73.3	0	0.0	5	16.7	2	6.7	1	3.3
		2015	27	81.8	0	0.0	4	12.1	1	3.0	1	3.0
		2016	14	41.2	1	2.9	13	38.2	5	14.7	1	2.9
		2017	27	64.3	1	2.4	8	19.0	5	11.9	1	2.4
		2018	21	58.3	1	2.8	6	16.7	7	19.4	1	2.8
	20–24	2010	15	100.0	0	0.0	-	-	-	-	-	-
		2011	14	93.3	1	6.7	-	-	-	-	-	-
		2012	22	84.6	2	7.7	0	0.0	2	7.7	0	0.0
		2013	10	66.7	2	13.3	0	0.0	3	20.0	0	0.0
		2014	20	83.3	1	4.2	1	4.2	1	4.2	1	4.2
		2015	11	64.7	0	0.0	4	23.5	2	11.8	0	0.0
		2016	18	60.0	0	0.0	7	23.3	4	13.3	1	3.3
		2017	13	35.1	0	0.0	15	40.5	9	24.3	0	0.0
		2018	12	54.5	0	0.0	6	27.3	4	18.2	0	0.0
	45–64	2010	19	86.4	3	13.6	-	-	-	-	-	-
		2011	22	88.0	3	12.0	-	-	-	-	-	-
		2012	21	72.4	2	6.9	2	6.9	3	10.3	1	3.4
		2013	8	42.1	4	21.1	1	5.3	5	26.3	1	5.3
		2014	12	80.0	0	0.0	2	13.3	1	6.7	0	0.0
		2015	12	46.2	0	0.0	10	38.5	4	15.4	0	0.0
		2016	6	16.7	1	2.8	19	52.8	10	28.6	0	0.0
		2017	26	37.1	1	1.4	20	28.6	23	33.3	0	0.0
		2018	11	22.0	0	0.0	25	50.0	12	24.0	2	4.0
	≥65	2010	4	44.4	5	55.6	-	-	-	-	-	-
		2011	12	85.7	2	14.3	-	-	-	-	-	-
		2012	12	66.7	1	5.6	1	5.6	4	22.2	0	0.0
		2013	2	15.4	1	7.7	4	30.8	6	46.2	0	0.0
		2014	7	36.8	0	0.0	5	26.3	7	36.8	0	0.0
		2015	12	34.3	0	0.0	11	31.4	12	34.3	0	0.0
		2016	5	10.0	0	0.0	30	60.0	15	30.0	0	0.0
		2017	10	15.2	2	3.0	31	47.0	22	33.3	1	1.5
		2018	10	25.6	1	2.6	17	43.6	11	28.2	0	0.0
Austria [7]	All ages	2010	47	55.3	27	31.8	4	4.7	4	4.7	3	3.5
		2011	15	30.6	8	16.3	1	2.0	2	4.1	23	46.9
		2012	26	46.4	12	21.4	1	1.8	2	3.6	15	26.8
		2013	34	60.7	15	26.8	3	5.4	4	7.1	0	0.0

Geographical Region	Age, y	Year	Serogroup B Cases n	Serogroup B Cases %	Serogroup C Cases n	Serogroup C Cases %	Serogroup W Cases n	Serogroup W Cases %	Serogroup Y Cases n	Serogroup Y Cases %	Other/NG Cases n	Other/NG Cases %
		2014	21	60.0	4	11.4	3	8.6	5	14.3	2	5.7
		2015	17	65.4	3	11.5	0	0.0	2	7.7	4	15.4
		2016	23	62.2	3	8.1	3	8.1	2	5.4	6	16.2
		2017	12	60.0	3	15.0	1	5.0	2	10.0	2	10.0
		2018	15	50.0	3	10.0	2	6.7	2	6.7	8	26.7
	<1	2010	8	72.7	2	18.2	0	0.0	0	0.0	1	9.1
		2011	1	20.0	2	40.0	0	0.0	1	20.0	1	20.0
		2012	3	42.9	2	28.6	0	0.0	0	0.0	2	28.6
		2013	11	91.7	1	8.3	0	0.0	0	0.0	0	0.0
		2014	4	100.0	0	0.0	0	0.0	0	0.0	0	0.0
		2015	7	77.8	1	11.1	0	0.0	0	0.0	1	11.1
		2016	4	66.7	0	0.0	0	0.0	0	0.0	2	33.3
		2017	5	83.3	0	0.0	0	0.0	0	0.0	1	16.7
		2018	1	50.0	0	0.0	0	0.0	1	50.0	0	0.0
	1–4	2010	7	41.2	9	52.9	1	5.9	0	0.0	0	0.0
		2011	2	40.0	0	0.0	0	0.0	0	0.0	3	60.0
		2012	7	63.6	2	18.2	0	0.0	0	0.0	2	18.2
		2013	5	62.5	3	37.5	0	0.0	0	0.0	0	0.0
		2014	3	50.0	2	33.3	0	0.0	1	16.7	0	0.0
		2015	3	100.0	0	0.0	0	0.0	0	0.0	0	0.0
		2016	3	75.0	0	0.0	0	0.0	0	0.0	1	25.0
		2017	1	50.0	0	0.0	0	0.0	0	0.0	1	50.0
		2018	2	50.0	0	0.0	0	0.0	0	0.0	2	50.0
	15–24	2010	14	50.0	9	32.1	2	7.1	2	7.1	1	3.6
		2011	6	37.5	3	18.8	0	0.0	0	0.0	7	43.8
		2012	10	50.0	4	20.0	0	0.0	1	5.0	5	25.0
		2013	5	41.7	6	50.0	0	0.0	1	8.3	0	0.0
		2014	5	41.7	1	8.3	2	16.7	2	16.7	2	16.7
		2015	3	60.0	1	20.0	0	0.0	0	0.0	1	20.0
		2016	8	53.3	1	6.7	3	20.0	1	6.7	2	13.3
		2017	1	25.0	2	50.0	0	0.0	1	25.0	0	0.0
		2018	3	75.0	0	0.0	0	0.0	0	0.0	1	25.0
	≥50	2010	5	41.7	4	33.3	1	8.3	1	8.3	1	8.3
		2011	1	9.1	1	9.1	1	9.1	0	0.0	8	72.7
		2012	3	60.0	1	20.0	0	0.0	0	0.0	1	20.0
		2013	3	37.5	1	12.5	2	25.0	2	25.0	0	0.0
		2014	6	75.0	1	12.5	0	0.0	1	12.5	0	0.0
		2015	2	66.7	0	0.0	0	0.0	1	33.3	0	0.0
		2016	3	75.0	0	0.0	0	0.0	1	25.0	0	0.0
		2017	3	60.0	1	20.0	0	0.0	1	20.0	0	0.0
		2018	5	62.5	0	0.0	1	12.5	1	12.5	1	12.5
Belgium [7]	All ages	2010	76	82.6	10	10.9	0	0.0	4	4.3	2	2.2
		2011	84	75.0	15	13.4	2	1.8	9	8.0	2	1.8
		2012	87	70.7	21	17.1	3	2.4	9	7.3	3	2.4
		2013	104	77.6	14	10.4	1	0.7	7	5.2	8	6.0
		2014	60	69.0	9	10.3	3	3.4	12	13.8	3	3.4
		2015	-	-	-	-	-	-	-	-	99	100.0
		2016	68	63.6	9	8.4	10	9.3	17	15.9	3	2.8
		2017	60	62.5	6	6.3	9	9.4	19	19.8	2	2.1
		2018	59	50.9	5	4.3	19	16.4	29	25.0	4	3.4
	<1	2010	10	83.3	1	8.3	0	0.0	1	8.3	0	0.0
		2011	14	87.5	2	12.5	0	0.0	0	0.0	0	0.0
		2012	16	84.2	3	15.8	0	0.0	0	0.0	0	0.0
		2013	13	86.7	1	6.7	0	0.0	0	0.0	1	6.7
		2014	11	73.3	2	13.3	1	6.7	1	6.7	0	0.0
		2015	-	-	-	-	-	-	-	-	19	100.0
		2016	10	76.9	0	0.0	2	15.4	1	7.7	0	0.0
		2017	9	69.2	1	7.7	1	7.7	2	15.4	0	0.0
		2018	4	66.7	0	0.0	1	16.7	1	16.7	0	0.0
	1–4	2010	16	100.0	0	0.0	0	0.0	0	0.0	0	0.0
		2011	22	91.7	0	0.0	0	0.0	0	0.0	2	8.3
		2012	24	82.8	3	10.3	0	0.0	1	3.4	1	3.4

Geographical Region	Age, y	Year	Serogroup B Cases n	Serogroup B Cases %	Serogroup C Cases n	Serogroup C Cases %	Serogroup W Cases n	Serogroup W Cases %	Serogroup Y Cases n	Serogroup Y Cases %	Other/NG Cases n	Other/NG Cases %
		2013	30	90.9	1	3.0	0	0.0	1	3.0	1	3.0
		2014	16	88.9	0	0.0	0	0.0	0	0.0	2	11.1
		2015	-	-	-	-	-	-	-	-	14	100.0
		2016	15	88.2	0	0.0	2	11.8	0	0.0	0	0.0
		2017	9	69.2	0	0.0	3	23.1	0	0.0	1	7.7
		2018	20	66.7	2	6.7	3	10.0	5	16.7	0	0.0
	15–24	2010	21	87.5	1	4.2	0	0.0	1	4.2	1	4.2
		2011	18	78.3	5	21.7	0	0.0	0	0.0	0	0.0
		2012	19	70.4	4	14.8	1	3.7	2	7.4	1	3.7
		2013	26	86.7	0	0.0	0	0.0	0	0.0	4	13.3
		2014	13	76.5	1	5.9	0	0.0	3	17.6	0	0.0
		2015	-	-	-	-	-	-	-	-	20	100.0
		2016	12	54.5	3	13.6	2	9.1	4	18.2	1	4.5
		2017	12	54.5	0	0.0	2	9.1	8	36.4	0	0.0
		2018	10	41.7	2	8.3	5	20.8	5	20.8	2	8.3
	≥50	2010	5	50.0	2	20.0	0	0.0	2	20.0	1	10.0
		2011	11	42.3	8	30.8	0	0.0	7	26.9	0	0.0
		2012	6	37.5	5	31.3	1	6.3	3	18.8	1	6.3
		2013	11	50.0	6	27.3	1	4.5	3	13.6	1	4.5
		2014	5	31.3	2	12.5	2	12.5	6	37.5	1	6.3
		2015	-	-	-	-	-	-	-	-	27	100.0
		2016	8	33.3	3	12.5	2	8.3	9	37.5	2	8.3
		2017	12	48.0	4	16.0	2	8.0	7	28.0	0	0.0
		2018	11	33.3	1	3.0	8	24.2	12	36.4	1	3.0
Brazil‡ [2,10,14,15]	All ages	2010	199	6.6	1213	40.5	76	2.5	14	0.5	1494	49.9
		2011	244	8.6	1145	40.4	84	3.0	31	1.1	1333	47.0
		2012	238	9.3	1037	40.7	66	2.6	38	1.5	1168	45.9
		2013	206	9.8	726	34.6	77	3.7	26	1.2	1063	50.7
		2014	149	9.3	549	34.1	59	3.7	23	1.4	830	51.6
		2015	158	12.1	360	27.6	53	4.1	13	1.0	718	55.1
		2016	117	10.5	313	28.1	47	4.2	17	1.5	620	55.7
		2017	146	12.9	363	32.0	53	4.7	16	1.4	557	49.1
		2018	167	15.0	304	27.2	50	4.5	17	1.5	578	51.8
	<1	2010	17	21.0	55	67.9	8	9.9	1	1.2	0	0.0
		2011	26	46.4	22	39.3	6	10.7	2	3.6	0	0.0
		2012	23	60.5	8	21.1	5	13.2	2	5.3	0	0.0
		2013	17	40.5	15	35.7	9	21.4	1	2.4	0	0.0
		2014	21	60.0	11	31.4	3	8.6	0	0.0	0	0.0
		2015	18	52.9	10	29.4	4	11.8	2	5.9	0	0.0
		2016	13	52.0	6	24.0	6	24.0	0	0.0	0	0.0
		2017	15	55.6	8	29.6	2	7.4	2	7.4	0	0.0
		2018	16	55.2	4	13.8	7	24.1	2	6.9	0	0.0
	1	2010	9	22.5	28	70.0	0	0.0	3	7.5	0	0.0
		2011	13	59.1	5	22.7	1	4.5	3	13.6	0	0.0
		2012	11	100.0	0	0.0	0	0.0	0	0.0	0	0.0
		2013	1	25.0	1	25.0	2	50.0	0	0.0	0	0.0
		2014	9	69.2	1	7.7	3	23.1	0	0.0	0	0.0
		2015	8	72.7	0	0.0	3	27.3	0	0.0	0	0.0
		2016	5	71.4	0	0.0	2	28.6	0	0.0	0	0.0
		2017	3	33.3	0	0.0	4	44.4	2	22.2	0	0.0
		2018	9	64.3	2	14.3	3	21.4	0	0.0	0	0.0
	2–4	2010	24	28.9	58	69.9	1	1.2	0	0.0	0	0.0
		2011	14	20.6	51	75.0	3	4.4	0	0.0	0	0.0
		2012	12	25.0	28	58.3	4	8.3	4	8.3	0	0.0
		2013	10	35.7	14	50.0	2	7.1	2	7.1	0	0.0
		2014	11	45.8	8	33.3	5	20.8	0	0.0	0	0.0
		2015	19	73.1	2	7.7	3	11.5	2	7.7	0	0.0
		2016	7	41.2	3	17.6	4	23.5	1	5.9	2	11.8
		2017	6	54.5	1	9.1	4	36.4	0	0.0	0	0.0
		2018	5	31.3	5	31.3	6	37.5	0	0.0	0	0.0
	15–29	2011	22	17.3	94	74.0	6	4.7	4	3.1	1	0.8
		2012	16	12.7	100	79.4	6	4.8	4	3.2	0	0.0

Geographical Region	Age, y	Year	Serogroup B Cases		Serogroup C Cases		Serogroup W Cases		Serogroup Y Cases		Other/NG Cases	
			n	%	n	%	n	%	n	%	n	%
		2013	12	15.0	64	80.0	3	3.8	1	1.3	0	0.0
		2014	10	13.9	54	75.0	5	6.9	3	4.2	0	0.0
		2015	6	13.3	35	77.8	3	6.7	1	2.2	0	0.0
		2016	5	11.1	35	77.8	1	2.2	4	8.9	0	0.0
		2017	7	15.6	32	71.1	4	8.9	2	4.4	0	0.0
		2018	10	16.9	41	69.5	6	10.2	2	3.4	0	0.0
	≥60	2011	1	2.9	29	82.9	2	5.7	2	5.7	1	2.9
		2012	3	9.7	20	64.5	4	12.9	4	12.9	0	0.0
		2013	2	11.8	10	58.8	0	0.0	4	23.5	1	5.9
		2014	0	0.0	8	50.0	2	12.5	6	37.5	0	0.0
		2015	1	8.3	6	50.0	4	33.3	1	8.3	0	0.0
		2016	4	25.0	5	31.3	3	18.8	2	12.5	2	12.5
		2017	1	5.3	11	57.9	5	26.3	2	10.5	0	0.0
		2018	2	8.7	14	60.9	5	21.7	2	8.7	0	0.0
Chile [16-19]	All ages	2012	38	32.5	3	2.6	60	51.3	2	1.7	14	12.0
		2013	46	33.8	2	1.5	85	62.5	0	0.0	3	2.2
		2014	31	22.1	2	1.4	102	72.9	1	0.7	4	2.9
		2015	40	33.3	1	0.8	69	57.5	2	1.7	8	6.7
		2016	31	29.2	2	1.9	69	65.1	1	0.9	3	2.8
		2017	26	32.5	1	1.3	47	58.8	1	1.3	5	6.3
		2018	32	42.1	1	1.3	38	50.0	2	2.6	3	3.9
		2019	32	46.4	4	5.8	30	43.5	3	4.3	0	0.0
	<0.75	2014	9	33.3	-	-	18	66.7	-	-	-	-
		2015	10	45.5	-	-	12	54.5	-	-	-	-
		2016	7	36.8	-	-	12	63.2	-	-	-	-
		2017	10	50.0	-	-	10	50.0	-	-	-	-
		2018	9	64.3	-	-	5	35.7	-	-	-	-
		2019	10	71.4	-	-	4	28.6	-	-	-	-
	<1	2012[§]	7	38.9	-	-	11	61.1	-	-	-	-
		2013	17	43.6	-	-	22	56.4	-	-	-	-
	0.75–1	2014	3	27.3	-	-	8	72.7	-	-	-	-
		2015	1	33.3	-	-	2	66.7	-	-	-	-
		2016	3	75.0	-	-	1	25.0	-	-	-	-
		2017	1	25.0	-	-	3	75.0	-	-	-	-
		2018	0	0.0	-	-	1	100.0	-	-	-	-
		2019	0	0.0	-	-	1	100.0	-	-	-	-
	1–4	2012[§]	0	0.0	-	-	10	100.0	-	-	-	-
		2013	8	100.0	-	-	0	0.0	-	-	-	-
		2014	8	61.5	-	-	5	38.5	-	-	-	-
		2015	7	100.0	-	-	0	0.0	-	-	-	-
		2016	4	80.0	-	-	1	20.0	-	-	-	-
		2017	3	100.0	-	-	0	0.0	-	-	-	-
		2018	5	100.0	-	-	0	0.0	-	-	-	-
		2019	6	85.7	-	-	1	14.3	-	-	-	-
	15–29	2012[§]	1	25.0	-	-	3	75.0	-	-	-	-
		2013	6	30.0	-	-	14	70.0	-	-	-	-
		2014	3	17.6	-	-	14	82.4	-	-	-	-
		2015	3	20.0	-	-	12	80.0	-	-	-	-
		2016	4	26.7	-	-	11	73.3	-	-	-	-
		2017	2	25.0	-	-	6	75.0	-	-	-	-
		2018	3	33.3	-	-	6	66.7	-	-	-	-
		2019	1	10.0	-	-	9	90.0	-	-	-	-
	≥60	2012[§]	2	25.0	-	-	6	75.0	-	-	-	-
		2013	2	9.1	-	-	20	90.9	-	-	-	-
		2014	3	10.0	-	-	27	90.0	-	-	-	-
		2015	4	16.7	-	-	20	83.3	-	-	-	-
		2016	4	16.7	-	-	20	83.3	-	-	-	-
		2017	4	22.2	-	-	14	77.8	-	-	-	-
		2018	6	33.3	-	-	12	66.7	-	-	-	-
		2019	8	61.5	-	-	5	38.5	-	-	-	-
Czech Republic [7]	All ages	2010	38	63.3	7	11.7	0	0.0	4	6.7	11	18.3
		2011	34	54.0	2	3.2	2	3.2	2	3.2	23	36.5

Geographical Region	Age, y	Year	Serogroup B Cases		Serogroup C Cases		Serogroup W Cases		Serogroup Y Cases		Other/NG Cases	
			n	%	n	%	n	%	n	%	n	%
		2012	45	76.3	8	13.6	1	1.7	1	1.7	4	6.8
		2013	43	72.9	7	11.9	0	0.0	2	3.4	7	11.9
		2014	24	57.1	9	21.4	2	4.8	1	2.4	6	14.3
		2015	31	64.6	10	20.8	3	6.3	1	2.1	3	6.3
		2016	24	55.8	10	23.3	4	9.3	1	2.3	4	9.3
		2017	32	47.8	25	37.3	3	4.5	1	1.5	6	9.0
		2018	23	41.1	24	42.9	4	7.1	3	5.4	2	3.6
	<1	2010	6	75.0	2	25.0	0	0.0	0	0.0	0	0.0
		2011	7	58.3	0	0.0	0	0.0	0	0.0	5	41.7
		2012	10	83.3	1	8.3	0	0.0	0	0.0	1	8.3
		2013	12	85.7	0	0.0	0	0.0	0	0.0	2	14.3
		2014	3	50.0	0	0.0	0	0.0	0	0.0	3	50.0
		2015	11	78.6	3	21.4	0	0.0	0	0.0	0	0.0
		2016	6	100.0	0	0.0	0	0.0	0	0.0	0	0.0
		2017	4	33.3	5	41.7	0	0.0	0	0.0	3	25.0
		2018	6	54.5	4	36.4	0	0.0	0	0.0	1	9.1
	1–4	2010	8	61.5	1	7.7	0	0.0	1	7.7	3	23.1
		2011	11	84.6	0	0.0	0	0.0	0	0.0	2	15.4
		2012	12	66.7	4	22.2	0	0.0	0	0.0	2	11.1
		2013	7	58.3	3	25.0	0	0.0	0	0.0	2	16.7
		2014	7	63.6	3	27.3	1	9.1	0	0.0	0	0.0
		2015	3	75.0	1	25.0	0	0.0	0	0.0	0	0.0
		2016	2	22.2	3	33.3	1	11.1	1	11.1	2	22.2
		2017	7	53.8	5	38.5	0	0.0	0	0.0	1	7.7
		2018	2	22.2	5	55.6	1	11.1	0	0.0	1	11.1
	15–24	2010	10	58.8	3	17.6	0	0.0	2	11.8	2	11.8
		2011	7	43.8	1	6.3	2	12.5	0	0.0	6	37.5
		2012	9	81.8	1	9.1	0	0.0	1	9.1	0	0.0
		2013	5	50.0	3	30.0	0	0.0	1	10.0	1	10.0
		2014	3	60.0	1	20.0	1	20.0	0	0.0	0	0.0
		2015	6	54.5	3	27.3	0	0.0	1	9.1	1	9.1
		2016	6	46.2	3	23.1	2	15.4	0	0.0	2	15.4
		2017	9	64.3	4	28.6	0	0.0	0	0.0	1	7.1
		2018	1	12.5	5	62.5	0	0.0	2	25.0	0	0.0
	≥50	2010	1	25.0	0	0.0	0	0.0	1	25.0	2	50.0
		2011	3	37.5	1	12.5	0	0.0	1	12.5	3	37.5
		2012	5	83.3	0	0.0	1	16.7	0	0.0	0	0.0
		2013	7	87.5	0	0.0	0	0.0	0	0.0	1	12.5
		2014	3	100.0	0	0.0	0	0.0	0	0.0	0	0.0
		2015	4	66.7	0	0.0	2	33.3	0	0.0	0	0.0
		2016	3	100.0	0	0.0	0	0.0	0	0.0	0	0.0
		2017	4	33.3	6	50.0	1	8.3	1	8.3	0	0.0
		2018	2	22.2	3	33.3	3	33.3	1	11.1	0	0.0
Denmark [7]	All ages	2010	36	54.5	26	39.4	0	0.0	1	1.5	3	4.5
		2011	27	37.5	39	54.2	1	1.4	5	6.9	0	0.0
		2012	19	33.9	30	53.6	1	1.8	6	10.7	0	0.0
		2013	20	36.4	16	29.1	0	0.0	4	7.3	15	27.3
		2014	21	46.7	7	15.6	2	4.4	5	11.1	10	22.2
		2015	10	45.5	6	27.3	2	9.1	4	18.2	0	0.0
		2016	15	39.5	4	10.5	9	23.7	3	7.9	7	18.4
		2017	10	25.6	1	2.6	14	35.9	8	20.5	6	15.4
		2018	18	50.0	6	16.7	9	25.0	2	5.6	1	2.8
	<1	2010	2	50.0	2	50.0	0	0.0	0	0.0	0	0.0
		2011	4	50.0	4	50.0	0	0.0	0	0.0	0	0.0
		2012	4	44.4	4	44.4	0	0.0	1	11.1	0	0.0
		2013	2	40.0	1	20.0	0	0.0	0	0.0	2	40.0
		2014	5	71.4	0	0.0	0	0.0	0	0.0	2	28.6
		2015	3	75.0	1	25.0	0	0.0	0	0.0	0	0.0
		2016	1	50.0	0	0.0	0	0.0	0	0.0	1	50.0
		2017	2	66.7	0	0.0	1	33.3	0	0.0	0	0.0
		2018	3	100.0	0	0.0	0	0.0	0	0.0	0	0.0
	1–4	2010	10	55.6	8	44.4	0	0.0	0	0.0	0	0.0

Geographical Region	Age, y	Year	Serogroup B Cases		Serogroup C Cases		Serogroup W Cases		Serogroup Y Cases		Other/NG Cases	
			n	%	n	%	n	%	n	%	n	%
		2011	5	35.7	9	64.3	0	0.0	0	0.0	0	0.0
		2012	7	53.8	6	46.2	0	0.0	0	0.0	0	0.0
		2013	6	75.0	1	12.5	0	0.0	0	0.0	1	12.5
		2014	3	60.0	0	0.0	1	20.0	0	0.0	1	20.0
		2015	1	50.0	1	50.0	0	0.0	0	0.0	0	0.0
		2016	4	100.0	0	0.0	0	0.0	0	0.0	0	0.0
		2017	1	33.3	0	0.0	0	0.0	0	0.0	2	66.7
		2018	5	62.5	2	25.0	1	12.5	0	0.0	0	0.0
	15–24	2010	11	55.0	9	45.0	0	0.0	0	0.0	0	0.0
		2011	7	41.2	7	41.2	0	0.0	3	17.6	0	0.0
		2012	2	33.3	3	50.0	0	0.0	1	16.7	0	0.0
		2013	7	41.2	5	29.4	0	0.0	2	11.8	3	17.6
		2014	2	33.3	2	33.3	0	0.0	0	0.0	2	33.3
		2015	0	0.0	2	33.3	1	16.7	3	50.0	0	0.0
		2016	3	33.3	1	11.1	2	22.2	1	11.1	2	22.2
		2017	3	30.0	1	10.0	2	20.0	1	10.0	3	30.0
		2018	1	20.0	1	20.0	1	20.0	1	20.0	1	20.0
	≥50	2010	6	42.9	5	35.7	0	0.0	1	7.1	2	14.3
		2011	5	27.8	11	61.1	1	5.6	1	5.6	0	0.0
		2012	6	27.3	11	50.0	1	4.5	4	18.2	0	0.0
		2013	1	10.0	5	50.0	0	0.0	1	10.0	3	30.0
		2014	8	44.4	2	11.1	0	0.0	5	27.8	3	16.7
		2015	3	50.0	1	16.7	1	16.7	1	16.7	0	0.0
		2016	4	22.2	2	11.1	7	38.9	2	11.1	3	16.7
		2017	2	11.1	0	0.0	9	50.0	7	38.9	0	0.0
		2018	5	41.7	1	8.3	6	50.0	0	0.0	0	0.0
Europe [7]	All ages	2010	2555	68.9	502	13.5	79	2.1	208	5.6	365	9.8
		2011	2573	67.1	506	13.2	83	2.2	288	7.5	385	10.0
		2012	2206	62.8	543	15.5	139	4.0	259	7.4	366	10.4
		2013	2143	62.2	467	13.5	147	4.3	287	8.3	404	11.7
		2014	1564	56.6	402	14.5	185	6.7	240	8.7	372	13.5
		2015	1685	54.0	404	12.9	317	10.2	291	9.3	426	13.6
		2016	1642	50.2	485	14.8	463	14.1	344	10.5	340	10.4
		2017	1530	47.4	485	15.0	512	15.9	347	10.8	352	10.9
		2018	1543	47.7	444	13.7	561	17.4	363	11.2	322	10.0
	<1	2010	580	83.5	44	6.3	4	0.6	11	1.6	56	8.1
		2011	535	81.4	49	7.5	5	0.8	11	1.7	57	8.7
		2012	460	77.8	59	10.0	12	2.0	8	1.4	52	8.8
		2013	484	80.0	35	5.8	12	2.0	18	3.0	56	9.3
		2014	385	75.2	43	8.4	22	4.3	15	2.9	47	9.2
		2015	360	70.0	41	8.0	40	7.8	20	3.9	53	10.3
		2016	301	68.9	42	9.6	34	7.8	17	3.9	43	9.8
		2017	272	63.3	52	12.1	45	10.5	10	2.3	51	11.9
		2018	286	66.8	40	9.3	56	13.1	15	3.5	31	7.2
	1–4	2010	690	77.6	101	11.4	10	1.1	10	1.1	78	8.8
		2011	702	79.8	75	8.5	13	1.5	15	1.7	75	8.5
		2012	618	75.1	66	8.0	24	2.9	9	1.1	106	12.9
		2013	530	78.1	54	8.0	11	1.6	8	1.2	76	11.2
		2014	391	73.6	44	8.3	15	2.8	5	0.9	76	14.3
		2015	427	71.8	54	9.1	37	6.2	11	1.8	66	11.1
		2016	395	69.5	41	7.2	54	9.5	7	1.2	71	12.5
		2017	343	64.6	52	9.8	53	10.0	16	3.0	67	12.6
		2018	342	67.7	44	8.7	42	8.3	14	2.8	63	12.5
	15–24	2010	469	63.3	130	17.5	20	2.7	61	8.2	61	8.2
		2011	478	61.8	140	18.1	17	2.2	63	8.2	75	9.7
		2012	400	59.2	131	19.4	28	4.1	56	8.3	61	9.0
		2013	362	58.3	112	18.0	37	6.0	52	8.4	58	9.3
		2014	242	55.0	69	15.7	39	8.9	38	8.6	52	11.8
		2015	259	47.2	88	16.0	61	11.1	61	11.1	80	14.6
		2016	293	48.1	101	16.6	90	14.8	64	10.5	61	10.0
		2017	278	49.6	75	13.4	76	13.6	73	13.0	58	10.4
		2018	266	49.8	77	14.4	68	12.7	63	11.8	60	11.2
	≥50	2010	279	50.7	75	13.6	38	6.9	86	15.6	72	13.1

Geographical Region	Age, y	Year	Serogroup B Cases		Serogroup C Cases		Serogroup W Cases		Serogroup Y Cases		Other/NG Cases	
			n	%	n	%	n	%	n	%	n	%
		2011	312	48.9	99	15.5	32	5.0	130	20.4	65	10.2
		2012	275	43.9	111	17.7	53	8.5	132	21.1	55	8.8
		2013	284	40.9	110	15.9	60	8.6	150	21.6	90	13.0
		2014	206	34.7	115	19.4	79	13.3	131	22.1	62	10.5
		2015	288	36.6	92	11.7	147	18.7	154	19.6	106	13.5
		2016	253	28.5	144	16.2	230	25.9	186	20.9	76	8.5
		2017	289	30.2	154	16.1	252	26.4	189	19.8	72	7.5
		2018	301	29.3	143	13.9	295	28.8	198	19.3	89	8.7
Finland [7]	All ages	2010	14	41.2	4	11.8	1	2.9	13	38.2	2	5.9
		2011	19	55.9	6	17.6	1	2.9	7	20.6	1	2.9
		2012	17	51.5	3	9.1	1	3.0	8	24.2	4	12.1
		2013	10	50.0	2	10.0	0	0.0	8	40.0	0	0.0
		2014	7	33.3	5	23.8	1	4.8	5	23.8	3	14.3
		2015	8	36.4	5	22.7	4	18.2	3	13.6	2	9.1
		2016	6	31.6	4	21.1	3	15.8	5	26.3	1	5.3
		2017	3	18.8	4	25.0	0	0.0	9	56.3	0	0.0
		2018	6	37.5	4	25.0	1	6.3	5	31.3	0	0.0
	<1	2010	3	75.0	0	0.0	0	0.0	1	25.0	0	0.0
		2011	1	50.0	0	0.0	0	0.0	0	0.0	1	50.0
		2012	3	100.0	0	0.0	0	0.0	0	0.0	0	0.0
		2013	5	100.0	0	0.0	0	0.0	0	0.0	0	0.0
		2014	3	75.0	1	25.0	0	0.0	0	0.0	0	0.0
		2015	1	100.0	0	0.0	0	0.0	0	0.0	0	0.0
		2016	1	100.0	0	0.0	0	0.0	0	0.0	0	0.0
		2017	1	100.0	0	0.0	0	0.0	0	0.0	0	0.0
		2018	0	0.0	1	100.0	0	0.0	0	0.0	0	0.0
	1–4	2010	3	60.0	2	40.0	0	0.0	0	0.0	0	0.0
		2011	3	100.0	0	0.0	0	0.0	0	0.0	0	0.0
		2012	1	50.0	0	0.0	1	50.0	0	0.0	0	0.0
		2013	3	60.0	2	40.0	0	0.0	0	0.0	0	0.0
		2014	1	100.0	0	0.0	0	0.0	0	0.0	0	0.0
		2015	2	40.0	1	20.0	0	0.0	0	0.0	2	40.0
		2016	0	0.0	0	0.0	0	0.0	0	0.0	0	0.0
		2017	0	0.0	0	0.0	0	0.0	0	0.0	0	0.0
		2018	1	100.0	0	0.0	0	0.0	0	0.0	0	0.0
	15–24	2010	4	40.0	0	0.0	1	10.0	4	40.0	1	10.0
		2011	10	76.9	2	15.4	0	0.0	1	7.7	0	0.0
		2012	7	70.0	0	0.0	0	0.0	2	20.0	1	10.0
		2013	0	0.0	0	0.0	0	0.0	0	0.0	0	0.0
		2014	1	20.0	2	40.0	0	0.0	1	20.0	1	20.0
		2015	1	14.3	3	42.9	1	14.3	2	28.6	0	0.0
		2016	1	16.7	1	16.7	1	16.7	2	33.3	1	16.7
		2017	1	25.0	0	0.0	0	0.0	3	75.0	0	0.0
		2018	1	25.0	1	25.0	0	0.0	2	50.0	0	0.0
	≥50	2010	1	14.3	0	0.0	0	0.0	5	71.4	1	14.3
		2011	2	22.2	3	33.3	0	0.0	4	44.4	0	0.0
		2012	4	33.3	2	16.7	0	0.0	4	33.3	2	16.7
		2013	0	0.0	0	0.0	0	0.0	6	100.0	0	0.0
		2014	2	28.6	1	14.3	0	0.0	3	42.9	1	14.3
		2015	3	50.0	1	16.7	1	16.7	1	16.7	0	0.0
		2016	3	30.0	2	20.0	2	20.0	3	30.0	0	0.0
		2017	1	11.1	2	22.2	0	0.0	6	66.7	0	0.0
		2018	3	50.0	0	0.0	0	0.0	3	50.0	0	0.0
France [7]	All ages	2010	369	72.2	82	16.0	11	2.2	27	5.3	22	4.3
		2011	395	70.2	84	14.9	14	2.5	45	8.0	25	4.4
		2012	366	66.5	99	18.0	40	7.3	30	5.5	15	2.7
		2013	325	56.5	147	25.6	27	4.7	56	9.7	20	3.5
		2014	230	54.8	122	29.0	19	4.5	40	9.5	9	2.1
		2015	242	52.4	119	25.8	32	6.9	54	11.7	15	3.2
		2016	261	51.0	134	26.2	45	8.8	62	12.1	10	2.0
		2017	226	41.5	149	27.3	74	13.6	78	14.3	18	3.3
		2018	217	49.4	93	21.2	62	14.1	57	13.0	10	2.3
	<1	2010	58	84.1	7	10.1	1	1.4	0	0.0	3	4.3

Geographical Region	Age, y	Year	Serogroup B Cases n	Serogroup B Cases %	Serogroup C Cases n	Serogroup C Cases %	Serogroup W Cases n	Serogroup W Cases %	Serogroup Y Cases n	Serogroup Y Cases %	Other/NG Cases n	Other/NG Cases %
		2011	70	80.5	9	10.3	1	1.1	3	3.4	4	4.6
		2012	52	74.3	12	17.1	3	4.3	1	1.4	2	2.9
		2013	53	66.3	16	20.0	3	3.8	5	6.3	3	3.8
		2014	44	62.9	19	27.1	1	1.4	3	4.3	3	4.3
		2015	41	63.1	14	21.5	3	4.6	6	9.2	1	1.5
		2016	45	59.2	24	31.6	4	5.3	3	3.9	0	0.0
		2017	45	64.3	15	21.4	6	8.6	1	1.4	3	4.3
		2018	44	73.3	4	6.7	10	16.7	2	3.3	0	0.0
	1–4	2010	89	80.9	12	10.9	0	0.0	4	3.6	5	4.5
		2011	91	80.5	9	8.0	1	0.9	6	5.3	6	5.3
		2012	74	77.9	8	8.4	11	11.6	0	0.0	2	2.1
		2013	55	78.6	8	11.4	2	2.9	2	2.9	3	4.3
		2014	41	73.2	13	23.2	1	1.8	0	0.0	1	1.8
		2015	43	66.2	17	26.2	4	6.2	1	1.5	0	0.0
		2016	49	71.0	11	15.9	3	4.3	4	5.8	2	2.9
		2017	39	59.1	10	15.2	11	16.7	5	7.6	1	1.5
		2018	41	69.5	11	18.6	6	10.2	0	0.0	1	1.7
	15–24	2010	88	68.2	26	20.2	2	1.6	10	7.8	3	2.3
		2011	98	65.8	31	20.8	3	2.0	12	8.1	5	3.4
		2012	98	66.2	30	20.3	6	4.1	10	6.8	4	2.7
		2013	75	55.6	41	30.4	8	5.9	8	5.9	3	2.2
		2014	48	58.5	22	26.8	3	3.7	7	8.5	2	2.4
		2015	53	51.0	26	25.0	9	8.7	11	10.6	5	4.8
		2016	47	48.5	28	28.9	12	12.4	9	9.3	1	1.0
		2017	40	39.2	29	28.4	14	13.7	15	14.7	4	3.9
		2018	37	42.0	26	29.5	11	12.5	11	12.5	3	3.4
	≥50	2010	48	69.6	8	11.6	6	8.7	6	8.7	1	1.4
		2011	52	57.1	15	16.5	5	5.5	17	18.7	2	2.2
		2012	53	48.2	24	21.8	14	12.7	16	14.5	3	2.7
		2013	45	36.0	35	28.0	6	4.8	31	24.8	8	6.4
		2014	35	39.8	26	29.5	9	10.2	17	19.3	1	1.1
		2015	43	38.7	29	26.1	10	9.0	24	21.6	5	4.5
		2016	44	32.6	36	26.7	19	14.1	33	24.4	3	2.2
		2017	47	27.3	49	28.5	33	19.2	39	22.7	4	2.3
		2018	37	33.9	17	15.6	20	18.3	31	28.4	4	3.7
Germany [7]	All ages	2010	237	61.4	77	19.9	11	2.8	19	4.9	42	10.9
		2011	227	61.5	65	17.6	8	2.2	18	4.9	51	13.8
		2012	207	58.5	79	22.3	13	3.7	13	3.7	42	11.9
		2013	212	61.4	56	16.2	8	2.3	24	7.0	45	13.0
		2014	175	63.4	42	15.2	11	4.0	18	6.5	30	10.9
		2015	182	63.4	41	14.3	11	3.8	21	7.3	32	11.1
		2016	163	49.4	59	17.9	26	7.9	32	9.7	50	15.2
		2017	141	49.5	38	13.3	25	8.8	35	12.3	46	16.1
		2018	141	48.8	34	11.8	33	11.4	36	12.5	45	15.6
	<1	2010	40	76.9	9	17.3	1	1.9	0	0.0	2	3.8
		2011	40	78.4	6	11.8	1	2.0	0	0.0	4	7.8
		2012	33	70.2	11	23.4	0	0.0	0	0.0	3	6.4
		2013	27	73.0	3	8.1	0	0.0	1	2.7	6	16.2
		2014	32	84.2	1	2.6	0	0.0	2	5.3	3	7.9
		2015	33	76.7	6	14.0	1	2.3	1	2.3	2	4.7
		2016	32	76.2	3	7.1	2	4.8	1	2.4	4	9.5
		2017	24	61.5	5	12.8	3	7.7	2	5.1	5	12.8
		2018	29	78.4	1	2.7	2	5.4	1	2.7	4	10.8
	1–4	2010	38	74.5	10	19.6	0	0.0	0	0.0	3	5.9
		2011	48	77.4	8	12.9	1	1.6	0	0.0	5	8.1
		2012	45	77.6	5	8.6	1	1.7	0	0.0	7	12.1
		2013	45	81.8	3	5.5	1	1.8	0	0.0	6	10.9
		2014	37	80.4	5	10.9	1	2.2	0	0.0	3	6.5
		2015	43	79.6	4	7.4	1	1.9	0	0.0	6	11.1
		2016	31	68.9	3	6.7	3	6.7	0	0.0	8	17.8
		2017	25	67.6	2	5.4	3	8.1	1	2.7	6	16.2
		2018	23	74.2	0	0.0	1	3.2	2	6.5	5	16.1
	15–24	2010	78	64.5	23	19.0	2	1.7	4	3.3	14	11.6

Geographical Region	Age, y	Year	Serogroup B Cases		Serogroup C Cases		Serogroup W Cases		Serogroup Y Cases		Other/NG Cases	
			n	%	n	%	n	%	n	%	n	%
		2011	61	56.0	26	23.9	3	2.8	4	3.7	15	13.8
		2012	47	55.3	21	24.7	7	8.2	2	2.4	8	9.4
		2013	52	58.4	21	23.6	2	2.2	7	7.9	7	7.9
		2014	37	60.7	8	13.1	2	3.3	6	9.8	8	13.1
		2015	40	55.6	9	12.5	5	6.9	7	9.7	11	15.3
		2016	40	51.3	10	12.8	5	6.4	15	19.2	8	10.3
		2017	33	57.9	7	12.3	5	8.8	8	14.0	4	7.0
		2018	28	46.7	4	6.7	6	10.0	7	11.7	15	25.0
	≥50	2010	24	34.3	14	20.0	7	10.0	10	14.3	15	21.4
		2011	26	41.9	10	16.1	2	3.2	13	21.0	11	17.7
		2012	34	45.3	15	20.0	3	4.0	10	13.3	13	17.3
		2013	33	42.9	15	19.5	5	6.5	13	16.9	11	14.3
		2014	27	39.1	20	29.0	6	8.7	6	8.7	10	14.5
		2015	39	52.7	11	14.9	3	4.1	10	13.5	11	14.9
		2016	26	28.0	29	31.2	11	11.8	13	14.0	14	15.1
		2017	32	34.4	13	14.0	11	11.8	19	20.4	18	19.4
		2018	27	29.3	16	17.4	14	15.2	21	22.8	14	15.2
Greece [7]	All ages	2010	41	74.5	1	1.8	0	0.0	0	0.0	13	23.6
		2011	44	84.6	2	3.8	0	0.0	0	0.0	6	11.5
		2012	43	72.9	0	0.0	1	1.7	3	5.1	12	20.3
		2013	43	72.9	2	3.4	2	3.4	1	1.7	11	18.6
		2014	38	63.3	7	11.7	2	3.3	2	3.3	11	18.3
		2015	40	74.1	0	0.0	0	0.0	4	7.4	10	18.5
		2016	38	73.1	0	0.0	1	1.9	2	3.8	11	21.2
		2017	26	61.9	1	2.4	2	4.8	2	4.8	11	26.2
		2018	25	73.5	5	14.7	1	2.9	2	5.9	1	2.9
	<1	2010	6	75.0	0	0.0	0	0.0	0	0.0	2	25.0
		2011	6	100.0	0	0.0	0	0.0	0	0.0	0	0.0
		2012	1	33.3	0	0.0	0	0.0	0	0.0	2	66.7
		2013	5	62.5	0	0.0	0	0.0	0	0.0	3	37.5
		2014	8	88.9	0	0.0	1	11.1	0	0.0	0	0.0
		2015	5	83.3	0	0.0	0	0.0	1	16.7	0	0.0
		2016	4	100.0	0	0.0	0	0.0	0	0.0	0	0.0
		2017	6	75.0	0	0.0	1	12.5	0	0.0	1	12.5
		2018	4	100.0	0	0.0	0	0.0	0	0.0	0	0.0
	1–4	2010	13	68.4	0	0.0	0	0.0	0	0.0	6	31.6
		2011	8	80.0	0	0.0	0	0.0	0	0.0	2	20.0
		2012	11	68.8	0	0.0	0	0.0	1	6.3	4	25.0
		2013	9	75.0	0	0.0	0	0.0	0	0.0	3	25.0
		2014	9	75.0	0	0.0	1	8.3	0	0.0	2	16.7
		2015	5	62.5	0	0.0	0	0.0	0	0.0	3	37.5
		2016	9	90.0	0	0.0	0	0.0	0	0.0	1	10.0
		2017	4	66.7	0	0.0	0	0.0	0	0.0	2	33.3
		2018	9	81.8	1	9.1	0	0.0	1	9.1	0	0.0
	15–24	2010	9	75.0	0	0.0	0	0.0	0	0.0	3	25.0
		2011	10	76.9	1	7.7	0	0.0	0	0.0	2	15.4
		2012	16	76.2	0	0.0	1	4.8	1	4.8	3	14.3
		2013	9	81.8	0	0.0	0	0.0	1	9.1	1	9.1
		2014	10	76.9	2	15.4	0	0.0	0	0.0	1	7.7
		2015	7	70.0	0	0.0	0	0.0	2	20.0	1	10.0
		2016	12	85.7	0	0.0	0	0.0	0	0.0	2	14.3
		2017	3	37.5	0	0.0	1	12.5	1	12.5	3	37.5
		2018	3	75.0	0	0.0	0	0.0	0	0.0	1	25.0
	≥50	2010	4	100.0	0	0.0	0	0.0	0	0.0	0	0.0
		2011	5	100.0	0	0.0	0	0.0	0	0.0	0	0.0
		2012	3	75.0	0	0.0	0	0.0	0	0.0	1	25.0
		2013	4	50.0	1	12.5	2	25.0	0	0.0	1	12.5
		2014	2	28.6	3	42.9	0	0.0	0	0.0	2	28.6
		2015	7	70.0	0	0.0	0	0.0	0	0.0	3	30.0
		2016	2	25.0	0	0.0	1	12.5	1	12.5	4	50.0
		2017	5	62.5	0	0.0	0	0.0	0	0.0	3	37.5
		2018	2	33.3	2	33.3	1	16.7	1	16.7	0	0.0
Ireland [7]	All ages	2010	93	94.9	4	4.1	1	1.0	0	0.0	0	0.0

Geographical Region	Age, y	Year	Serogroup B Cases		Serogroup C Cases		Serogroup W Cases		Serogroup Y Cases		Other/NG Cases	
			n	%	n	%	n	%	n	%	n	%
		2011	84	94.4	2	2.2	1	1.1	1	1.1	1	1.1
		2012	58	96.7	0	0.0	0	0.0	2	3.3	0	0.0
		2013	70	90.9	1	1.3	2	2.6	2	2.6	2	2.6
		2014	66	86.8	6	7.9	1	1.3	2	2.6	1	1.3
		2015	44	64.7	11	16.2	5	7.4	7	10.3	1	1.5
		2016	48	56.5	22	25.9	7	8.2	5	5.9	3	3.5
		2017	28	39.4	28	39.4	12	16.9	1	1.4	2	2.8
		2018	46	52.3	22	25.0	12	13.6	8	9.1	0	0.0
	<1	2010	28	100.0	0	0.0	0	0.0	0	0.0	0	0.0
		2011	29	100.0	0	0.0	0	0.0	0	0.0	0	0.0
		2012	17	100.0	0	0.0	0	0.0	0	0.0	0	0.0
		2013	24	96.0	0	0.0	0	0.0	1	4.0	0	0.0
		2014	18	90.0	0	0.0	1	5.0	1	5.0	0	0.0
		2015	11	84.6	1	7.7	1	7.7	0	0.0	0	0.0
		2016	10	55.6	4	22.2	3	16.7	1	5.6	0	0.0
		2017	6	42.9	5	35.7	3	21.4	0	0.0	0	0.0
		2018	6	50.0	5	41.7	1	8.3	0	0.0	0	0.0
	1–4	2010	34	97.1	0	0.0	1	2.9	0	0.0	0	0.0
		2011	26	96.3	0	0.0	1	3.7	0	0.0	0	0.0
		2012	20	100.0	0	0.0	0	0.0	0	0.0	0	0.0
		2013	19	95.0	0	0.0	0	0.0	0	0.0	1	5.0
		2014	24	100.0	0	0.0	0	0.0	0	0.0	0	0.0
		2015	11	84.6	1	7.7	1	7.7	0	0.0	0	0.0
		2016	12	80.0	0	0.0	3	20.0	0	0.0	0	0.0
		2017	10	66.7	3	20.0	1	6.7	0	0.0	1	6.7
		2018	13	72.2	3	16.7	2	11.1	0	0.0	0	0.0
	15–24	2010	11	91.7	1	8.3	0	0.0	0	0.0	0	0.0
		2011	9	100.0	0	0.0	0	0.0	0	0.0	0	0.0
		2012	7	87.5	0	0.0	0	0.0	1	12.5	0	0.0
		2013	11	91.7	0	0.0	1	8.3	0	0.0	0	0.0
		2014	8	80.0	2	20.0	0	0.0	0	0.0	0	0.0
		2015	5	38.5	5	38.5	1	7.7	2	15.4	0	0.0
		2016	13	65.0	6	30.0	0	0.0	0	0.0	1	5.0
		2017	1	9.1	5	45.5	4	36.4	0	0.0	1	9.1
		2018	7	36.8	5	26.3	3	15.8	4	21.1	0	0.0
	≥50	2010	8	88.9	1	11.1	0	0.0	0	0.0	0	0.0
		2011	3	50.0	2	33.3	0	0.0	0	0.0	1	16.7
		2012	3	75.0	0	0.0	0	0.0	1	25.0	0	0.0
		2013	7	87.5	0	0.0	0	0.0	0	0.0	1	12.5
		2014	3	50.0	3	50.0	0	0.0	0	0.0	0	0.0
		2015	4	36.4	2	18.2	1	9.1	3	27.3	1	9.1
		2016	4	26.7	6	40.0	1	6.7	2	13.3	2	13.3
		2017	4	21.1	11	57.9	3	15.8	1	5.3	0	0.0
		2018	7	36.8	6	31.6	4	21.1	2	10.5	0	0.0
Italy [7]	All ages	2010	74	49.3	21	14.0	3	2.0	11	7.3	41	27.3
		2011	75	49.3	19	12.5	4	2.6	16	10.5	38	25.0
		2012	54	40.0	32	23.7	1	0.7	18	13.3	30	22.2
		2013	49	30.2	35	21.6	3	1.9	17	10.5	58	35.8
		2014	50	32.1	35	22.4	7	4.5	15	9.6	49	31.4
		2015	47	25.1	57	30.5	7	3.7	22	11.8	54	28.9
		2016	69	30.3	81	35.5	13	5.7	25	11.0	40	17.5
		2017	74	37.6	57	28.9	12	6.1	34	17.3	20	10.2
		2018	70	41.2	43	25.3	15	8.8	28	16.5	14	8.2
	<1	2010	18	69.2	4	15.4	0	0.0	0	0.0	4	15.4
		2011	10	55.6	2	11.1	0	0.0	1	5.6	5	27.8
		2012	9	56.3	1	6.3	0	0.0	1	6.3	5	31.3
		2013	9	52.9	3	17.6	0	0.0	1	5.9	4	23.5
		2014	14	66.7	3	14.3	0	0.0	1	4.8	3	14.3
		2015	10	50.0	4	20.0	2	10.0	1	5.0	3	15.0
		2016	13	59.1	1	4.5	2	9.1	2	9.1	4	18.2
		2017	11	73.3	1	6.7	0	0.0	1	6.7	2	13.3
		2018	7	58.3	2	16.7	0	0.0	2	16.7	1	8.3
	1–4	2010	14	51.9	3	11.1	0	0.0	0	0.0	10	37.0

Geographical Region	Age, y	Year	Serogroup B Cases		Serogroup C Cases		Serogroup W Cases		Serogroup Y Cases		Other/NG Cases	
			n	%	n	%	n	%	n	%	n	%
		2011	15	65.2	4	17.4	0	0.0	1	4.3	3	13.0
		2012	11	44.0	4	16.0	0	0.0	1	4.0	9	36.0
		2013	14	58.3	5	20.8	1	4.2	1	4.2	3	12.5
		2014	10	41.7	1	4.2	1	4.2	2	8.3	10	41.7
		2015	7	38.9	2	11.1	0	0.0	1	5.6	8	44.4
		2016	7	31.8	6	27.3	1	4.5	1	4.5	7	31.8
		2017	9	47.4	4	21.1	1	5.3	3	15.8	2	10.5
		2018	10	58.8	2	11.8	2	11.8	0	0.0	3	17.6
	15–24	2010	7	35.0	5	25.0	1	5.0	1	5.0	6	30.0
		2011	16	50.0	5	15.6	0	0.0	2	6.3	9	28.1
		2012	8	36.4	7	31.8	0	0.0	3	13.6	4	18.2
		2013	11	44.0	3	12.0	0	0.0	2	8.0	9	36.0
		2014	3	16.7	5	27.8	3	16.7	1	5.6	6	33.3
		2015	8	19.5	16	39.0	2	4.9	4	9.8	11	26.8
		2016	14	26.9	18	34.6	2	3.8	5	9.6	13	25.0
		2017	17	50.0	5	14.7	2	5.9	9	26.5	1	2.9
		2018	15	50.0	10	33.3	3	10.0	0	0.0	2	6.7
	≥50	2010	8	33.3	1	4.2	1	4.2	4	16.7	10	41.7
		2011	10	37.0	4	14.8	2	7.4	2	7.4	9	33.3
		2012	10	35.7	6	21.4	0	0.0	6	21.4	6	21.4
		2013	7	15.9	8	18.2	1	2.3	4	9.1	24	54.5
		2014	10	22.7	16	36.4	1	2.3	8	18.2	9	20.5
		2015	9	17.3	13	25.0	2	3.8	12	23.1	16	30.8
		2016	13	26.5	23	46.9	3	6.1	3	6.1	7	14.3
		2017	15	24.6	23	37.7	4	6.6	12	19.7	7	11.5
		2018	15	24.2	20	32.3	6	9.7	14	22.6	7	11.3
The Netherlands [7]	All ages	2010	104	72.7	10	7.0	5	3.5	10	7.0	14	9.8
		2011	79	74.5	4	3.8	1	0.9	13	12.3	9	8.5
		2012	76	69.1	4	3.6	2	1.8	15	13.6	13	11.8
		2013	79	73.1	5	4.6	6	5.6	10	9.3	8	7.4
		2014	56	67.5	3	3.6	1	1.2	12	14.5	11	13.3
		2015	65	72.2	8	8.9	9	10.0	7	7.8	1	1.1
		2016	77	50.7	6	3.9	51	33.6	17	11.2	1	0.7
		2017	81	40.9	9	4.5	80	40.4	27	13.6	1	0.5
		2018	74	35.9	3	1.5	103	50.0	24	11.7	2	1.0
	<1	2010	23	92.0	2	8.0	0	0.0	0	0.0	0	0.0
		2011	13	86.7	0	0.0	0	0.0	0	0.0	2	13.3
		2012	16	84.2	2	10.5	0	0.0	0	0.0	1	5.3
		2013	14	93.3	0	0.0	1	6.7	0	0.0	0	0.0
		2014	8	80.0	0	0.0	0	0.0	1	10.0	1	10.0
		2015	9	75.0	2	16.7	1	8.3	0	0.0	0	0.0
		2016	14	100.0	0	0.0	0	0.0	0	0.0	0	0.0
		2017	11	68.8	1	6.3	4	25.0	0	0.0	0	0.0
		2018	9	60.0	0	0.0	5	33.3	1	6.7	0	0.0
	1–4	2010	29	82.9	1	2.9	1	2.9	1	2.9	3	8.6
		2011	24	92.3	0	0.0	0	0.0	0	0.0	2	7.7
		2012	25	78.1	1	3.1	0	0.0	3	9.4	3	9.4
		2013	22	91.7	0	0.0	0	0.0	0	0.0	2	8.3
		2014	15	93.8	0	0.0	0	0.0	0	0.0	1	6.3
		2015	10	100.0	0	0.0	0	0.0	0	0.0	0	0.0
		2016	12	75.0	0	0.0	4	25.0	0	0.0	0	0.0
		2017	17	81.0	0	0.0	4	19.0	0	0.0	0	0.0
		2018	22	88.0	0	0.0	3	12.0	0	0.0	0	0.0
	15–24	2010	19	65.5	2	6.9	2	6.9	1	3.4	5	17.2
		2011	14	58.3	2	8.3	1	4.2	5	20.8	2	8.3
		2012	12	66.7	0	0.0	1	5.6	1	5.6	4	22.2
		2013	11	84.6	0	0.0	1	7.7	0	0.0	1	7.7
		2014	10	71.4	1	7.1	0	0.0	2	14.3	1	7.1
		2015	18	85.7	1	4.8	1	4.8	0	0.0	1	4.8
		2016	16	51.6	1	3.2	11	35.5	3	9.7	0	0.0
		2017	24	53.3	1	2.2	17	37.8	2	4.4	1	2.2
		2018	15	41.7	0	0.0	15	41.7	6	16.7	0	0.0

Geographical Region	Age, y	Year	Serogroup B Cases		Serogroup C Cases		Serogroup W Cases		Serogroup Y Cases		Other/NG Cases	
			n	%	n	%	n	%	n	%	n	%
	≥50	2010	14	51.9	2	7.4	1	3.7	6	22.2	4	14.8
		2011	15	71.4	2	9.5	0	0.0	3	14.3	1	4.8
		2012	10	55.6	0	0.0	1	5.6	6	33.3	1	5.6
		2013	15	48.4	3	9.7	4	12.9	7	22.6	2	6.5
		2014	8	34.8	2	8.7	1	4.3	6	26.1	6	26.1
		2015	18	54.5	3	9.1	6	18.2	6	18.2	0	0.0
		2016	17	27.0	3	4.8	31	49.2	11	17.5	1	1.6
		2017	12	14.6	6	7.3	39	47.6	25	30.5	0	0.0
		2018	19	18.8	2	2.0	63	62.4	15	14.9	2	2.0
New Zealand¶ [20]	All ages	2018	51	43.6	10	8.5	33	28.2	16	13.7	7	6.0
		2019	62	46.3	7	5.2	36	26.9	16	11.9	13	9.7
	<1	2018	11	64.7	1	5.9	3	17.6	1	5.9	1	5.9
		2019	16	51.6	0	0.0	8	25.8	1	3.2	6	19.4
	1–4	2018	7	46.7	1	6.7	4	26.7	1	6.7	2	13.3
		2019	10	47.6	0	0.0	5	23.8	1	4.8	5	23.8
	15–19	2018	12	63.2	2	10.5	3	15.8	0	0.0	2	10.5
		2019	9	60.0	2	13.3	2	13.3	1	6.7	1	6.7
	20–29	2018	8	53.3	1	6.7	5	33.3	1	6.7	0	0.0
		2019	6	35.3	1	5.9	8	47.1	1	5.9	1	5.9
	60–69	2018	1	11.1	0	0.0	3	33.3	5	55.6	0	0.0
		2019	3	27.3	1	9.1	3	27.3	4	36.4	0	0.0
	≥70	2018	0	0.0	1	9.1	4	36.4	6	54.5	0	0.0
		2019	2	16.7	0	0.0	5	41.7	4	33.3	1	8.3
Norway [7]	All ages	2010	13	33.3	10	25.6	2	5.1	13	33.3	1	2.6
		2011	10	27.0	4	10.8	2	5.4	20	54.1	1	2.7
		2012	9	37.5	9	37.5	0	0.0	6	25.0	0	0.0
		2013	11	40.7	6	22.2	0	0.0	7	25.9	3	11.1
		2014	7	38.9	1	5.6	3	16.7	5	27.8	2	11.1
		2015	8	42.1	2	10.5	0	0.0	8	42.1	1	5.3
		2016	9	37.5	0	0.0	6	25.0	9	37.5	0	0.0
		2017	3	16.7	2	11.1	5	27.8	8	44.4	0	0.0
		2018	9	34.6	1	3.8	4	15.4	12	46.2	0	0.0
	<1	2010	0	0.0	1	100.0	0	0.0	0	0.0	0	0.0
		2011	3	100.0	0	0.0	0	0.0	0	0.0	0	0.0
		2012	2	66.7	1	33.3	0	0.0	0	0.0	0	0.0
		2013	2	66.7	0	0.0	0	0.0	0	0.0	1	33.3
		2014	1	50.0	0	0.0	0	0.0	0	0.0	1	50.0
		2015	0	0.0	0	0.0	0	0.0	0	0.0	0	0.0
		2016	2	66.7	0	0.0	1	33.3	0	0.0	0	0.0
		2017	0	0.0	0	0.0	0	0.0	0	0.0	0	0.0
		2018	0	0.0	0	0.0	0	0.0	0	0.0	0	0.0
	1–4	2010	2	33.3	2	33.3	1	16.7	0	0.0	1	16.7
		2011	4	66.7	2	33.3	0	0.0	0	0.0	0	0.0
		2012	1	100.0	0	0.0	0	0.0	0	0.0	0	0.0
		2013	3	75.0	1	25.0	0	0.0	0	0.0	0	0.0
		2014	3	100.0	0	0.0	0	0.0	0	0.0	0	0.0
		2015	3	100.0	0	0.0	0	0.0	0	0.0	0	0.0
		2016	2	100.0	0	0.0	0	0.0	0	0.0	0	0.0
		2017	1	100.0	0	0.0	0	0.0	0	0.0	0	0.0
		2018	4	80.0	0	0.0	0	0.0	1	20.0	0	0.0
	15–24	2010	7	33.3	6	28.6	0	0.0	8	38.1	0	0.0
		2011	0	0.0	0	0.0	0	0.0	7	100.0	0	0.0
		2012	4	30.8	4	30.8	0	0.0	5	38.5	0	0.0
		2013	1	20.0	0	0.0	0	0.0	3	60.0	1	20.0
		2014	1	50.0	1	50.0	0	0.0	0	0.0	0	0.0
		2015	0	0.0	2	33.3	0	0.0	4	66.7	0	0.0
		2016	1	33.3	0	0.0	0	0.0	2	66.7	0	0.0
		2017	0	0.0	0	0.0	1	20.0	4	80.0	0	0.0
		2018	1	16.7	1	16.7	0	0.0	4	66.7	0	0.0
	≥50	2010	2	28.6	1	14.3	1	14.3	3	42.9	0	0.0
		2011	2	18.2	1	9.1	2	18.2	5	45.5	1	9.1
		2012	1	25.0	2	50.0	0	0.0	1	25.0	0	0.0

Geographical Region	Age, y	Year	Serogroup B Cases n	%	Serogroup C Cases n	%	Serogroup W Cases n	%	Serogroup Y Cases n	%	Other/NG Cases n	%
		2013	2	33.3	2	33.3	0	0.0	2	33.3	0	0.0
		2014	2	25.0	0	0.0	1	12.5	4	50.0	1	12.5
		2015	2	40.0	0	0.0	0	0.0	3	60.0	0	0.0
		2016	3	23.1	0	0.0	4	30.8	6	46.2	0	0.0
		2017	1	11.1	2	22.2	3	33.3	3	33.3	0	0.0
		2018	2	25.0	0	0.0	1	12.5	5	62.5	0	0.0
Poland [7]	All ages	2010	114	50.0	92	40.4	1	0.4	5	2.2	16	7.0
		2011	152	53.9	96	34.0	2	0.7	2	0.7	30	10.6
		2012	129	54.2	90	37.8	4	1.7	2	0.8	13	5.5
		2013	136	54.4	55	22.0	3	1.2	1	0.4	55	22.0
		2014	82	43.9	44	23.5	1	0.5	3	1.6	57	30.5
		2015	145	66.2	50	22.8	7	3.2	7	3.2	10	4.6
		2016	106	63.5	42	25.1	7	4.2	2	1.2	10	6.0
		2017	135	59.7	53	23.5	9	4.0	2	0.9	27	11.9
		2018	120	60.3	41	20.6	17	8.5	3	1.5	18	9.0
	<1	2010	38	71.7	10	18.9	0	0.0	0	0.0	5	9.4
		2011	35	64.8	10	18.5	0	0.0	1	1.9	8	14.8
		2012	33	63.5	15	28.8	0	0.0	0	0.0	4	7.7
		2013	35	68.6	6	11.8	0	0.0	0	0.0	10	19.6
		2014	26	57.8	9	20.0	0	0.0	0	0.0	10	22.2
		2015	40	87.0	4	8.7	2	4.3	0	0.0	0	0.0
		2016	33	80.5	5	12.2	2	4.9	1	2.4	0	0.0
		2017	34	72.3	5	10.6	3	6.4	0	0.0	5	10.6
		2018	32	71.1	6	13.3	7	15.6	0	0.0	0	0.0
	1–4	2010	39	53.4	31	42.5	1	1.4	0	0.0	2	2.7
		2011	40	51.3	26	33.3	0	0.0	0	0.0	12	15.4
		2012	33	56.9	20	34.5	2	3.4	0	0.0	3	5.2
		2013	36	53.7	16	23.9	0	0.0	0	0.0	15	22.4
		2014	23	50.0	7	15.2	0	0.0	0	0.0	16	34.8
		2015	37	64.9	16	28.1	2	3.5	0	0.0	2	3.5
		2016	32	71.1	9	20.0	1	2.2	0	0.0	3	6.7
		2017	34	65.4	14	26.9	1	1.9	0	0.0	3	5.8
		2018	35	72.9	8	16.7	4	8.3	0	0.0	1	2.1
	15–24	2010	12	33.3	19	52.8	0	0.0	3	8.3	2	5.6
		2011	29	49.2	22	37.3	2	3.4	0	0.0	6	10.2
		2012	20	54.1	14	37.8	1	2.7	1	2.7	1	2.7
		2013	29	55.8	13	25.0	0	0.0	0	0.0	10	19.2
		2014	10	40.0	6	24.0	0	0.0	1	4.0	8	32.0
		2015	12	44.4	10	37.0	1	3.7	4	14.8	0	0.0
		2016	11	52.4	8	38.1	1	4.8	0	0.0	1	4.8
		2017	17	50.0	8	23.5	1	2.9	1	2.9	7	20.6
		2018	10	38.5	11	42.3	0	0.0	0	0.0	5	19.2
	≥50	2010	8	42.1	7	36.8	0	0.0	1	5.3	3	15.8
		2011	16	59.3	10	37.0	0	0.0	0	0.0	1	3.7
		2012	12	48.0	11	44.0	0	0.0	0	0.0	2	8.0
		2013	16	50.0	6	18.8	3	9.4	1	3.1	6	18.8
		2014	4	19.0	9	42.9	1	4.8	1	4.8	6	28.6
		2015	21	58.3	5	13.9	2	5.6	2	5.6	6	16.7
		2016	11	42.3	7	26.9	3	11.5	1	3.8	4	15.4
		2017	22	57.9	10	26.3	2	5.3	1	2.6	3	7.9
		2018	18	58.1	5	16.1	2	6.5	3	9.7	3	9.7
Portugal [7]	All ages	2010	48	60.8	6	7.6	0	0.0	0	0.0	25	31.6
		2011	53	68.8	2	2.6	0	0.0	10	13.0	12	15.6
		2012	44	63.8	4	5.8	1	1.4	4	5.8	16	23.2
		2013	48	78.7	2	3.3	1	1.6	3	4.9	7	11.5
		2014	34	65.4	2	3.8	0	0.0	4	7.7	12	23.1
		2015	47	72.3	4	6.2	0	0.0	7	10.8	7	10.8
		2016	28	73.7	0	0.0	2	5.3	4	10.5	4	10.5
		2017	32	65.3	2	4.1	1	2.0	4	8.2	10	20.4
		2018	41	71.9	5	8.8	5	8.8	5	8.8	1	1.8
	<1	2010	12	75.0	0	0.0	0	0.0	0	0.0	4	25.0
		2011	18	90.0	0	0.0	0	0.0	0	0.0	2	10.0
		2012	11	68.8	0	0.0	0	0.0	0	0.0	5	31.3

Geographical Region	Age, y	Year	Serogroup B Cases		Serogroup C Cases		Serogroup W Cases		Serogroup Y Cases		Other/NG Cases	
			n	%	n	%	n	%	n	%	n	%
		2013	22	100.0	0	0.0	0	0.0	0	0.0	0	0.0
		2014	9	75.0	1	8.3	0	0.0	0	0.0	2	16.7
		2015	8	88.9	0	0.0	0	0.0	1	11.1	0	0.0
		2016	5	62.5	0	0.0	0	0.0	1	12.5	2	25.0
		2017	9	81.8	0	0.0	0	0.0	1	9.1	1	9.1
		2018	16	88.9	0	0.0	1	5.6	1	5.6	0	0.0
	1–4	2010	12	57.1	0	0.0	0	0.0	0	0.0	9	42.9
		2011	12	70.6	0	0.0	0	0.0	3	17.6	2	11.8
		2012	21	87.5	0	0.0	0	0.0	0	0.0	3	12.5
		2013	10	90.9	0	0.0	0	0.0	0	0.0	1	9.1
		2014	10	66.7	0	0.0	0	0.0	1	6.7	4	26.7
		2015	11	84.6	0	0.0	0	0.0	0	0.0	2	15.4
		2016	9	100.0	0	0.0	0	0.0	0	0.0	0	0.0
		2017	8	72.7	0	0.0	0	0.0	3	27.3	0	0.0
		2018	12	100.0	0	0.0	0	0.0	0	0.0	0	0.0
	15–24	2010	5	71.4	0	0.0	0	0.0	0	0.0	2	28.6
		2011	5	50.0	0	0.0	0	0.0	3	30.0	2	20.0
		2012	2	22.2	2	22.2	0	0.0	1	11.1	4	44.4
		2013	5	71.4	1	14.3	0	0.0	1	14.3	0	0.0
		2014	2	66.7	0	0.0	0	0.0	0	0.0	1	33.3
		2015	6	60.0	2	20.0	0	0.0	1	10.0	1	10.0
		2016	1	100.0	0	0.0	0	0.0	0	0.0	0	0.0
		2017	1	50.0	1	50.0	0	0.0	0	0.0	0	0.0
		2018	3	75.0	0	0.0	0	0.0	1	25.0	0	0.0
	≥50	2010	7	50.0	2	14.3	0	0.0	0	0.0	5	35.7
		2011	6	66.7	1	11.1	0	0.0	1	11.1	1	11.1
		2012	1	25.0	0	0.0	1	25.0	0	0.0	2	50.0
		2013	5	41.7	1	8.3	1	8.3	1	8.3	4	33.3
		2014	2	25.0	1	12.5	0	0.0	2	25.0	3	37.5
		2015	9	56.3	2	12.5	0	0.0	2	12.5	3	18.8
		2016	6	66.7	0	0.0	1	11.1	0	0.0	2	22.2
		2017	7	53.8	0	0.0	1	7.7	0	0.0	5	38.5
		2018	3	30.0	2	20.0	3	30.0	1	10.0	1	10.0
Spain [7]	All Ages	2010	295	73.0	52	12.9	8	2.0	2	0.5	47	11.6
		2011	308	71.5	67	15.5	8	1.9	4	0.9	44	10.2
		2012	211	63.0	54	16.1	13	3.9	5	1.5	52	15.5
		2013	193	73.7	24	9.2	6	2.3	6	2.3	33	12.6
		2014	105	71.9	13	8.9	2	1.4	4	2.7	22	15.1
		2015	142	67.6	20	9.5	8	3.8	6	2.9	34	16.2
		2016	151	57.6	23	8.8	13	5.0	18	6.9	57	21.8
		2017	139	51.9	26	9.7	27	10.1	18	6.7	58	21.6
		2018	156	39.8	48	12.2	70	17.9	47	12.0	71	18.1
	<1	2010	64	85.3	1	1.3	0	0.0	0	0.0	10	13.3
		2011	65	89.0	2	2.7	0	0.0	0	0.0	6	8.2
		2012	49	84.5	1	1.7	1	1.7	0	0.0	7	12.1
		2013	46	92.0	0	0.0	1	2.0	1	2.0	2	4.0
		2014	37	94.9	0	0.0	0	0.0	0	0.0	2	5.1
		2015	27	81.8	0	0.0	3	9.1	1	3.0	2	6.1
		2016	31	79.5	1	2.6	1	2.6	0	0.0	6	15.4
		2017	25	78.1	1	3.1	2	6.3	0	0.0	4	12.5
		2018	24	63.2	1	2.6	4	10.5	1	2.6	8	21.1
	1–4	2010	93	84.5	5	4.5	2	1.8	1	0.9	9	8.2
		2011	87	87.0	4	4.0	1	1.0	1	1.0	7	7.0
		2012	58	75.3	0	0.0	2	2.6	2	2.6	15	19.5
		2013	54	81.8	1	1.5	1	1.5	2	3.0	8	12.1
		2014	24	77.4	2	6.5	0	0.0	0	0.0	5	16.1
		2015	32	82.1	1	2.6	0	0.0	0	0.0	6	15.4
		2016	42	79.2	0	0.0	0	0.0	0	0.0	11	20.8
		2017	34	69.4	1	2.0	2	4.1	2	4.1	10	20.4
		2018	36	58.1	2	3.2	6	9.7	2	3.2	16	25.8
	15–24	2010	32	64.0	6	12.0	2	4.0	1	2.0	9	18.0
		2011	29	61.7	12	25.5	0	0.0	1	2.1	5	10.6
		2012	16	39.0	14	34.1	1	2.4	1	2.4	9	22.0

Geographical Region	Age, y	Year	Serogroup B Cases		Serogroup C Cases		Serogroup W Cases		Serogroup Y Cases		Other/NG Cases	
			n	%	n	%	n	%	n	%	n	%
		2013	13	65.0	5	25.0	1	5.0	0	0.0	1	5.0
		2014	7	46.7	2	13.3	1	6.7	2	13.3	3	20.0
		2015	13	72.2	1	5.6	0	0	0	0.0	4	22.2
		2016	13	43.3	4	13.3	3	10.0	3	10.0	7	23.3
		2017	15	42.9	4	11.4	4	11.4	7	20.0	5	14.3
		2018	15	38.5	6	15.4	8	20.5	5	12.8	5	12.8
	≥50	2010	39	59.1	16	24.2	4	6.1	0	0.0	7	10.6
		2011	48	60.8	16	20.3	5	6.3	1	1.3	9	11.4
		2012	38	54.3	15	21.4	5	7.1	1	1.4	11	15.7
		2013	39	60.9	9	14.1	3	4.7	3	4.7	10	15.6
		2014	18	64.3	4	14.3	1	3.6	1	3.6	4	14.3
		2015	37	56.9	10	15.4	4	6.2	4	6.2	10	15.4
		2016	27	37.0	8	11.0	9	12.3	10	13.7	19	26.0
		2017	38	40.0	11	11.6	15	15.8	7	7.4	24	25.3
		2018	54	31.0	20	11.5	41	23.6	29	16.7	30	17.2
Sweden [7]	All ages	2010	15	22.4	24	35.8	5	7.5	23	34.3	0	0.0
		2011	15	22.1	14	20.6	1	1.5	32	47.1	6	8.8
		2012	23	22.3	26	25.2	5	4.9	45	43.7	4	3.9
		2013	19	25.7	11	14.9	3	4.1	39	52.7	2	2.7
		2014	7	14.6	18	37.5	2	4.2	17	35.4	4	8.3
		2015	13	25.0	7	13.5	10	19.2	17	32.7	5	9.6
		2016	10	16.1	10	16.1	18	29.0	18	29.0	6	9.7
		2017	7	14.3	7	14.3	17	34.7	14	28.6	4	8.2
		2018	5	8.9	8	14.3	21	37.5	15	26.8	7	12.5
	<1	2010	0	0.0	0	0.0	0	0.0	1	100.0	0	0.0
		2011	2	40.0	2	40.0	0	0.0	0	0.0	1	20.0
		2012	1	100.0	0	0.0	0	0.0	0	0.0	0	0.0
		2013	1	50.0	0	0.0	0	0.0	1	50.0	0	0.0
		2014	2	50.0	1	25.0	0	0.0	1	25.0	0	0.0
		2015	0	0.0	0	0.0	0	0.0	0	0.0	1	100.0
		2016	2	100.0	0	0.0	0	0.0	0	0.0	0	0.0
		2017	1	50.0	1	50.0	0	0.0	0	0.0	0	0.0
		2018	1	33.3	1	33.3	0	0.0	1	33.3	0	0.0
	1–4	2010	3	37.5	4	50.0	1	12.5	0	0.0	0	0.0
		2011	4	50.0	2	25.0	0	0.0	1	12.5	1	12.5
		2012	3	37.5	5	62.5	0	0.0	0	0.0	0	0.0
		2013	2	50.0	2	50.0	0	0.0	0	0.0	0	0.0
		2014	2	100.0	0	0.0	0	0.0	0	0.0	0	0.0
		2015	3	60.0	1	20.0	0	0.0	1	20.0	0	0.0
		2016	0	0.0	3	60.0	1	20.0	0	0.0	1	20.0
		2017	2	50.0	0	0.0	2	50.0	0	0.0	0	0.0
		2018	0	0.0	1	33.3	0	0.0	0	0.0	2	66.7
	15–24	2010	2	10.0	11	55.0	3	15.0	4	20.0	0	0.0
		2011	6	31.6	5	26.3	0	0.0	8	42.1	0	0.0
		2012	6	28.6	5	23.8	2	9.5	7	33.3	1	4.8
		2013	5	41.7	2	16.7	0	0.0	5	41.7	0	0.0
		2014	1	14.3	5	71.4	0	0.0	1	14.3	0	0.0
		2015	3	33.3	2	22.2	3	33.3	0	0.0	1	11.1
		2016	4	21.1	4	21.1	7	36.8	3	15.8	1	5.3
		2017	1	8.3	2	16.7	6	50.0	2	16.7	1	8.3
		2018	1	9.1	0	0.0	3	27.3	5	45.5	2	18.2
	≥50	2010	5	19.2	5	19.2	1	3.8	15	57.7	0	0.0
		2011	0	0.0	3	13.0	0	0.0	18	78.3	2	8.7
		2012	5	9.1	12	21.8	3	5.5	32	58.2	3	5.5
		2013	7	15.9	5	11.4	3	6.8	28	63.6	1	2.3
		2014	1	3.7	7	25.9	2	7.4	15	55.6	2	7.4
		2015	5	16.7	1	3.3	6	20.0	16	53.3	2	6.7
		2016	2	7.4	1	3.7	10	37.0	13	48.1	1	3.7
		2017	0	0.0	2	11.1	4	22.2	10	55.6	2	11.1
		2018	3	8.8	5	14.7	15	44.1	9	26.5	2	5.9
United Kingdom [7]	All ages	2010	857	85.0	20	2.0	25	2.5	70	6.9	36	3.6
		2011	837	80.8	29	2.8	34	3.3	96	9.3	40	3.9

Geographical Region	Age, y	Year	Serogroup B Cases		Serogroup C Cases		Serogroup W Cases		Serogroup Y Cases		Other/NG Cases	
			n	%	n	%	n	%	n	%	n	%
		2012	674	78.2	32	3.7	50	5.8	89	10.3	17	2.0
		2013	616	72.3	33	3.9	82	9.6	91	10.7	30	3.5
		2014	483	64.4	38	5.1	122	16.3	87	11.6	20	2.7
		2015	527	56.4	36	3.9	216	23.1	120	12.8	36	3.9
		2016	452	52.6	46	5.4	236	27.5	116	13.5	9	1.0
		2017	411	53.2	55	7.1	217	28.1	82	10.6	8	1.0
		2018	436	56.1	68	8.8	181	23.3	82	10.6	10	1.3
	<1	2010	246	93.2	1	0.4	2	0.8	7	2.7	8	3.0
		2011	203	91.0	4	1.8	3	1.3	5	2.2	8	3.6
		2012	176	90.3	1	0.5	8	4.1	5	2.6	5	2.6
		2013	164	87.2	0	0.0	7	3.7	8	4.3	9	4.8
		2014	137	83.0	2	1.2	18	10.9	4	2.4	4	2.4
		2015	125	74.0	1	0.6	27	16.0	9	5.3	7	4.1
		2016	65	71.4	1	1.1	17	18.7	5	5.5	3	3.3
		2017	51	61.4	9	10.8	19	22.9	3	3.6	1	1.2
		2018	69	62.7	11	10.0	25	22.7	4	3.6	1	0.9
	1–4	2010	259	95.2	4	1.5	2	0.7	3	1.1	4	1.5
		2011	267	91.1	4	1.4	8	2.7	2	0.7	12	4.1
		2012	239	94.5	2	0.8	7	2.8	1	0.4	4	1.6
		2013	186	92.1	2	1.0	6	3.0	2	1.0	6	3.0
		2014	144	92.3	1	0.6	8	5.1	1	0.6	2	1.3
		2015	175	80.3	2	0.9	27	12.4	8	3.7	6	2.8
		2016	139	79.4	1	0.6	32	18.3	1	0.6	2	1.1
		2017	108	77.1	5	3.6	25	17.9	1	0.7	1	0.7
		2018	87	81.3	4	3.7	13	12.1	3	2.8	0	0.0
	15–24	2010	122	80.8	1	0.7	5	3.3	19	12.6	4	2.6
		2011	122	81.9	1	0.7	6	4.0	15	10.1	5	3.4
		2012	86	73.5	6	5.1	7	6.0	16	13.7	2	1.7
		2013	79	60.3	6	4.6	24	18.3	18	13.7	4	3.1
		2014	70	59.8	4	3.4	26	22.2	12	10.3	5	4.3
		2015	71	50.4	3	2.1	36	25.5	20	14.2	11	7.8
		2016	87	60.0	5	3.4	36	24.8	15	10.3	2	1.4
		2017	90	73.2	2	1.6	17	13.8	10	8.1	4	3.3
		2018	108	80.0	1	0.7	13	9.6	9	6.7	4	3.0
	≥50	2010	86	58.9	4	2.7	15	10.3	31	21.2	10	6.8
		2011	96	55.2	6	3.4	14	8.0	54	31.0	4	2.3
		2012	69	46.3	5	3.4	23	15.4	48	32.2	4	2.7
		2013	66	42.6	10	6.5	29	18.7	47	30.3	3	1.9
		2014	57	31.1	10	5.5	55	30.1	56	30.6	5	2.7
		2015	69	26.3	10	3.8	109	41.6	68	26.0	6	2.3
		2016	66	23.0	20	7.0	123	42.9	78	27.2	0	0.0
		2017	68	26.7	14	5.5	123	48.2	50	19.6	0	0.0
		2018	75	27.9	36	13.4	107	39.8	49	18.2	2	0.7
United States [6]	All ages	2015	161	44.8	54	15.0	37	10.3	43	12.0	64	17.8
		2016	130	34.9	99	26.6	34	9.1	52	14.0	57	15.3
		2017	134	38.3	86	24.6	26	7.4	31	8.9	73	20.9
		2018	119	36.2	90	27.4	17	5.2	48	14.6	55	16.7
	<1	2015	31	72.1	5	11.6	0	0.0	2	4.7	5	11.6
		2016	26	72.2	4	11.1	1	2.8	1	2.8	4	11.1
		2017	15	60.0	0	0.0	2	8.0	2	8.0	6	24.0
		2018	21	65.6	6	18.8	2	6.3	1	3.1	2	6.3
	1–4	2015	16	51.6	8	25.8	3	9.7	0	0.0	4	12.9
		2016	15	45.5	9	27.3	3	9.1	5	15.2	1	3.0
		2017	9	45.0	5	25.0	0	0.0	0	0.0	6	30.0
		2018	12	41.4	10	34.5	1	3.4	4	13.8	2	6.9
	16–23	2015	28	58.3	3	6.3	3	6.3	4	8.3	10	20.8
		2016	41	56.9	11	15.3	2	2.8	5	6.9	13	18.1
		2017	48	69.6	2	2.9	2	2.9	3	4.3	14	20.3
		2018	21	61.8	3	8.8	0	0.0	0	0.0	10	29.4
	45–64	2015	32	41.0	6	7.7	14	17.9	11	14.1	15	19.2
		2016	11	16.2	26	38.2	11	16.2	13	19.1	7	10.3
		2017	14	20.3	30	43.5	7	10.1	5	7.2	13	18.8

Geographical Region	Age, y	Year	Serogroup B Cases		Serogroup C Cases		Serogroup W Cases		Serogroup Y Cases		Other/NG Cases	
			n	%	n	%	n	%	n	%	n	%
		2018	25	31.6	22	27.8	7	8.9	14	17.7	11	13.9
	≥65	2015	12	19.7	11	18.0	10	16.4	18	29.5	10	16.4
		2016	9	14.8	12	19.7	11	18.0	19	31.1	10	16.4
		2017	21	28.0	16	21.3	8	10.7	15	20.0	15	20.0
		2018	16	21.6	23	31.1	4	5.4	19	25.7	12	16.2

IMD=invasive meningococcal disease; NG=nongroupable.
A dash indicates no incidence data were available.

*Percentages were calculated from available data.

†No age-stratified data specific for W, Y, NG, or other serogroups were available in 2010 or 2011, so percentages were calculated using the total number of serogroup B and C cases as the denominator.

‡All-ages serogroup data were sourced from Ministério da Saúde do Brasil [2] and included cases with 'ignored serogroups', which are shown here as other/NG cases. Age-stratified data were sourced from regional SIREVA II reports for 2010–2016 [10] and Adolfo Lutz Institute reports for 2017–2018 [14,15].

§2012 age group serogroup data only span through to week 43; single C isolates were reported for the <1 y and 15–29 y age groups.

¶For all-ages data, other/NG cases include serogroup X and E isolates, NG isolates, and laboratory confirmed cases that did not undergo serogrouping; for age-stratified data, other/NG cases additionally include probable cases (ie, clinically compatible illnesses that were not laboratory confirmed).

Supplementary Figure S1. Number of cases of invasive meningococcal disease (A) worldwide and (B) in selected European countries by serogroup from 2010 to 2019 (countries with available data) [1,2,4-13,16-25]. Data from China, Colombia, Dominican Republic, Japan, Kuwait, Mozambique, Paraguay, South Korea, Uruguay, and Venezuela are not shown. For Russia only, serogroups are shown as percentages of total cases. *2017 data are from January to June. †Other represents all other cases, including serogroup A. ‡NG represents NG isolates as well as cases for which a serogroup was not determined. §NG represents cases with unknown or ignored serogroup. ¶2011 data only span through Week 47. #NG represents all other cases, including serogroup A. ||Other/NG represents all other cases, including serogroup A. NG=nongroupable.

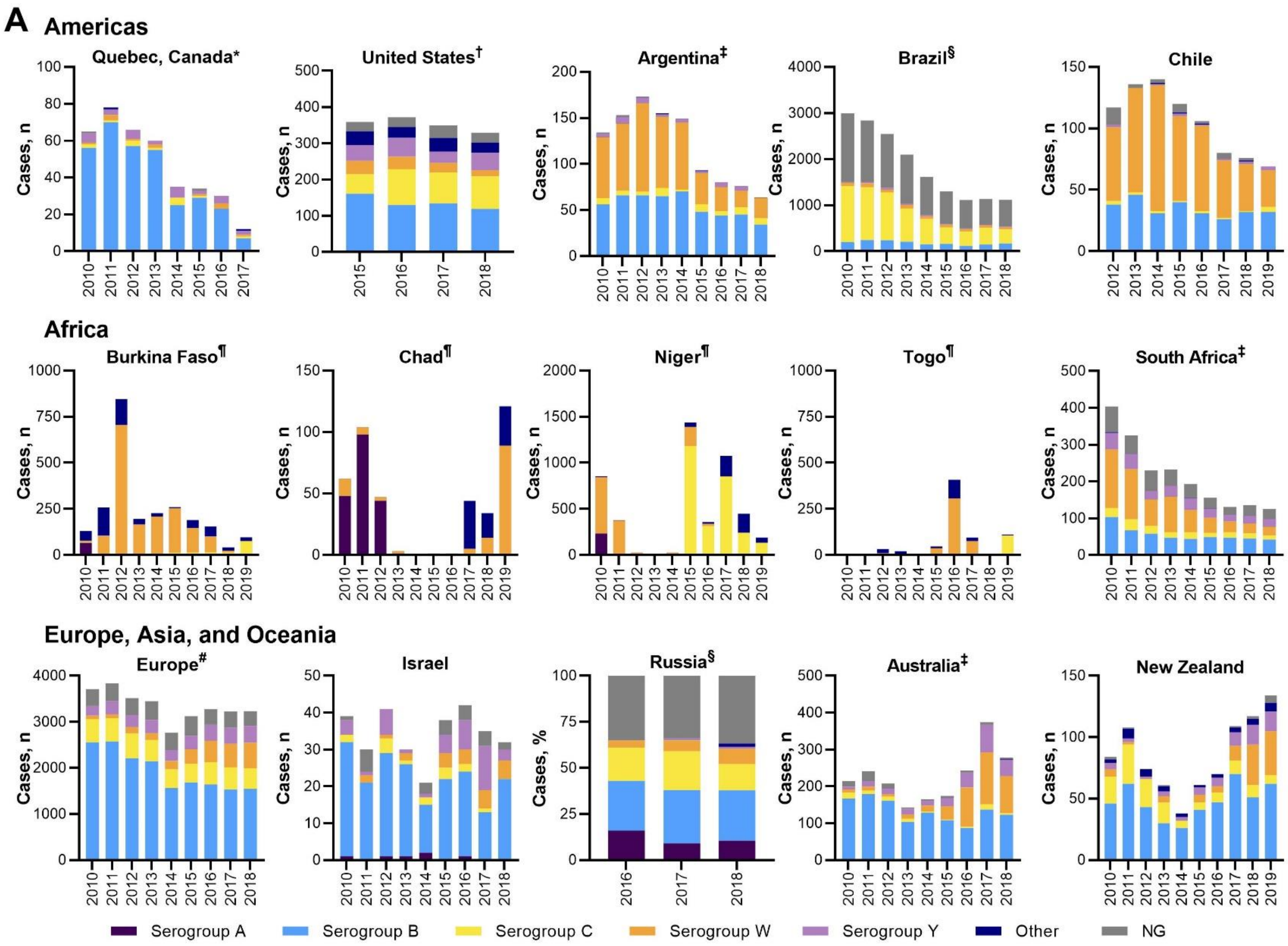
A
Americas
Quebec, Canada*
United States†
Argentina‡
Brazil§
Chile
Africa
Burkina Faso¶
Chad¶
Niger¶
Togo¶
South Africa‡
Europe, Asia, and Oceania
Europe#
Israel
Russia§
Australia‡
New Zealand
Cases, n
Cases, %
Serogroup A
Serogroup B
Serogroup C
Serogroup W
Serogroup Y
Other
NG

B

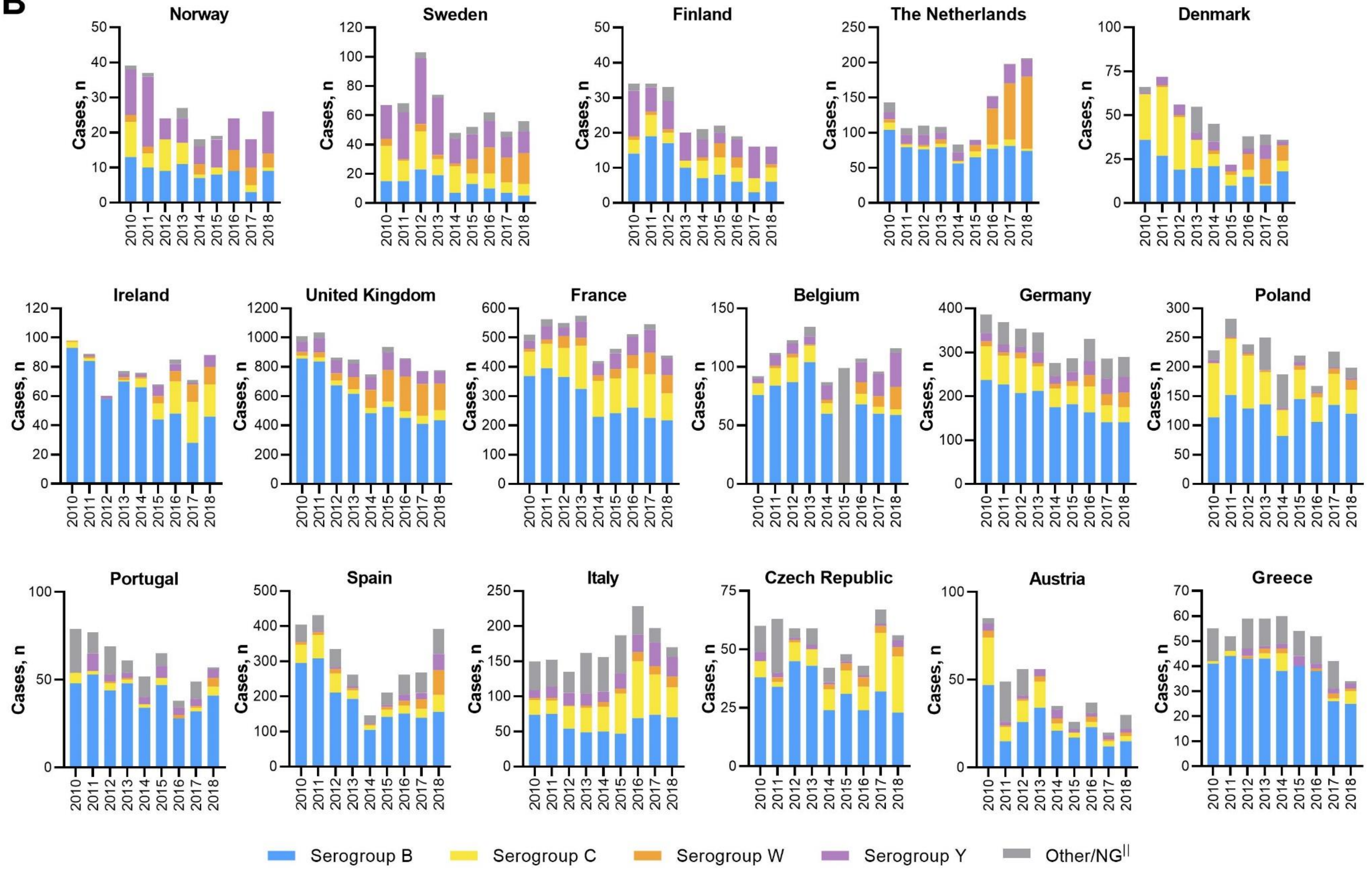
Norway
Sweden
Finland
The Netherlands
Denmark
Ireland
United Kingdom
France
Belgium
Germany
Poland
Portugal
Spain
Italy
Czech Republic
Austria
Greece
Cases, n
Serogroup B
Serogroup C
Serogroup W
Serogroup Y
Other/NG||

References

1. **World Health Organization. Menigitis Weekly Bulletin Week, 2010-2019** (https://www.who.int/publications/m/item/meningitis-week-48-52-29-november-2010---2-january-2011; https://www.who.int/publications/m/item/meningitis-week-44-47-31-october---27-november-2011; https://www.who.int/publications/m/item/meningitis-week-49-52-3---30-december-2012; https://cdn.who.int/media/docs/default-source/bulletins-2013/bulletinmeningite2013_s49_52.pdf; https://cdn.who.int/media/docs/default-source/bulletins-2014/bulletinmeningite_s49_52_2014.pdf; https://www.who.int/publications/m/item/meningitis-weekly-bulletin-30-november-2015---3-january-2016; https://cdn.who.int/media/docs/default-source/medicines/bulletins-2016/bulletin_meningite_s48_52_2016.pdf; https://www.who.int/publications/m/item/meningitis-weekly-bulletin-4---31-december-2017; https://www.who.int/publications/m/item/meningitis-weekly-bulletin-3---30-december-2018; https://www.who.int/publications/m/item/meningitis-weekly-bulleint-29-december-2019). Accessed, November 18, 2022.

2. **Ministério da Saúde do Brasil. Casos Confirmados, Óbitos, Incidência (por 100.000 Habitantes) e Letalidade (%) por Tipo de Meningite. Brasil, 2010 a 2018** (https://www.gov.br/saude/pt-br/assuntos/saude-de-a-a-z/m/meningite-1 (downloadable during conduct of research)). Accessed November 11, 2021.

3. **Public Health Agency of Canada. Notifiable Disease Charts** (https://dsol-smed.phac-aspc.gc.ca/notifiable/charts-list). Accessed November 11, 2021.

4. **New Zealand Ministry of Health. The Epidemiology of Meningococcal Disease in New Zealand 2010-2013** (https://surv.esr.cri.nz/PDF_surveillance/MeningococcalDisease/2010/2010AnnualRpt.pdf; https://surv.esr.cri.nz/PDF_surveillance/MeningococcalDisease/2011/2011AnnualRpt.pdf; https://surv.esr.cri.nz/PDF_surveillance/MeningococcalDisease/2012/2012AnnualRpt.pdf; https://surv.esr.cri.nz/PDF_surveillance/MeningococcalDisease/2013/2013AnnualRpt.pdf). Accessed November 18, 2021.

5. **New Zealand Ministry of Health. Notifiable Diseases in New Zealand Annual Report 2014-2018** (https://surv.esr.cri.nz/PDF_surveillance/AnnualRpt/AnnualSurv/2014/2014AnnualReportFinal.pdf; https://surv.esr.cri.nz/PDF_surveillance/AnnualRpt/AnnualSurv/2015/2015AnnualReportFinal.pdf; https://surv.esr.cri.nz/PDF_surveillance/AnnualRpt/AnnualSurv/2016/2016AnnualNDReportFinal.pdf; https://surv.esr.cri.nz/PDF_surveillance/AnnualRpt/AnnualSurv/2017/2017AnnualNDReport_FINAL.pdf; https://surv.esr.cri.nz/PDF_surveillance/AnnualRpt/AnnualSurv/2018/2018AnnualNDReport_FINAL.pdf). Accessed November, 18, 2021.

6. **Centers for Disease Control and Prevention. Enhanced Meningococcal Disease Surveillance Report, 2015-2018** (https://www.cdc.gov/meningococcal/downloads/NCIRD-EMS-Report-2015.pdf; https://www.cdc.gov/meningococcal/downloads/NCIRD-EMS-Report.pdf; https://www.cdc.gov/meningococcal/downloads/NCIRD-EMS-Report-2017.pdf; https://www.cdc.gov/meningococcal/downloads/NCIRD-EMS-Report-2018.pdf). Accessed November, 18, 2021.

7. **European Centre for Disease Prevention and Control. Surveillance Atlas of Infectious Diseases** (https://atlas.ecdc.europa.eu/public/index.aspx?Dataset=27&HealthTopic=36). Accessed October 8, 2021.

8. **Organización Panamericana de la Salud. Informe Regional de SIREVA II, 2017** (https://iris.paho.org/handle/10665.2/53136#:~:text=Esta%20publicaci%C3%B3n%20proporci

ona%20informaci%C3%B3n%20actualizada,de%20pa%C3%ADses%20de%20las%20Am%C3%A9ricas). Accessed July 13, 2022.

9. **Organización Panamericana de la Salud. Informe Regional de SIREVA II, 2018** (https://iris.paho.org/handle/10665.2/54567). Accessed June 18, 2022.

10. **Organización Panamericana de la Salud. Informe Regional de SIREVA II, 2010-2016** (https://www.paho.org/hq/dmdocuments/2011/SIREVA-II-2010.pdf; https://www.paho.org/hq/dmdocuments/2012/SIREVA-II-2011-Sp.pdf; https://www3.paho.org/hq/index.php?option=com_docman&view=download&category_slug=servicios-laboratorio-1732&alias=22372-informe-regional-sireva-ii-2012-372&Itemid=270&lang=en; http://iris.paho.org/xmlui/bitstream/handle/123456789/31147/9789275319185-spa.pdf; https://iris.paho.org/bitstream/handle/10665.2/33875/9789275319420-spa.pdf; https://iris.paho.org/bitstream/handle/10665.2/49091/9789275320099_spa.pdf; https://iris.paho.org/bitstream/handle/10665.2/51781/9789275321850_spa.pdf?sequence=1&isAllowed=y). Accessed November 18, 2021.

11. **Australian Meningococcal Surveillance Programme. Australian Meningococcal Surveillance Programme Annual Report, 2017** (https://doi.org/10.33321/cdi.2019.43.66). Accessed June 14, 2022.

12. **Australian Meningococcal Surveillance Programme. Australian Meningococcal Surveillance Programme Annual Report, 2018** (https://www.researchgate.net/publication/339954493). Accessed June 14, 2022.

13. **Australian Meningococcal Surveillance Programme. Australian Meningococcal Surveillance Programme Annual Report, 2010-2016** (https://www1.health.gov.au/internet/main/publishing.nsf/Content/cda-cdi3503-pdf-cnt.htm/$FILE/cdi3503a.pdf; https://www1.health.gov.au/internet/main/publishing.nsf/Content/cda-cdi3603-pdf-cnt.htm/$FILE/cdi3603c.pdf; https://www1.health.gov.au/internet/main/publishing.nsf/Content/cda-cdi3703-pdf-cnt.htm/$FILE/cdi3703e.pdf; (https://www1.health.gov.au/internet/main/publishing.nsf/Content/cda-cdi3804-pdf-cnt.htm/$FILE/cdi3804f.pdf; https://www1.health.gov.au/internet/main/publishing.nsf/Content/cda-cdi4002-pdf-cnt.htm/$FILE/cdi4002f.pdf; https://www1.health.gov.au/internet/main/publishing.nsf/Content/cda-cdi4004-pdf-cnt.htm/$FILE/cdi4004i.pdf; https://www1.health.gov.au/internet/main/publishing.nsf/Content/52063507BA07755FCA25823300191F9B/$File/cdi4104-i.pdf). Accessed November 18, 2021.

14. **Secretaria De Estado Da Saúde, Coordenadoria De Controle De Doenças, Instituto Adolfo Lutz. Informação da Vigilância das Pneumonias e Meningites Bacterianas, 2017** (http://www.ial.sp.gov.br/resources/insituto-adolfo-lutz/publicacoes/sireva_2017_2.pdf). Accessed November 18, 2021.

15. **Secretaria De Estado Da Saúde, Coordenadoria De Controle De Doenças, Instituto Adolfo Lutz. Informação da Vigilância das Pneumonias e Meningites Bacterianas, 2018** (http://www.ial.sp.gov.br/resources/insituto-adolfo-lutz/publicacoes/sireva_2018_atualizado.pdf). Accessed November 18, 2021.

16. **Instituto de Salud Pública de Chile. Informe de Resultados de Vigilancia de Laboratorio Enfermedad Invasora *Neisseria meningitidis* 2011-2012** (https://www.ispch.cl/sites/default/files/neisseria_meningitidis_44.pdf). Accessed August 5, 2022.

17. **Instituto de Salud Pública de Chile. Informe de Resultados de Vigilancia de Laboratorio Enfermedad Invasora *Neisseria meningitidis* 2013**

(https://www.ispch.cl/sites/default/files/Informe%20Neisseria%20meningitidis%20%20SE%2052%202013.pdf). Accessed August 17, 2022.

18. **Instituto de Salud Pública de Chile. Informe de Resultados de Vigilancia de Laboratorio Enfermedad Invasora *Neisseria meningitidis* 2014** (https://www.ispch.cl/sites/default/files/Informe%20Neisseria%20meningitidis%20%20SE%201-53%202014.pdf). Accessed August 17, 2022.

19. **Instituto de Salud Pública de Chile. Informe de Resultados de Vigilancia de Laboratorio Enfermedad Invasora Neisseria meningitidis 2015-2019** (https://www.ispch.cl/sites/default/files/Informe_Neisseria_meningitidis%20_SE_24_0.pdf; https://www.ispch.cl/sites/default/files/Informe_Neisseria_meningitidis%20_2016_v3.pdf; https://www.ispch.cl/sites/default/files/Informe%20Neisseria%20meningitidis%20%20SE%201-52%202017%20v2.pdf; https://www.ispch.cl/sites/default/files/Informe%20Neisseria%20meningitidis%20%20SE%201-52%202018.pdf; https://www.ispch.cl/sites/default/files/Informe%20Neisseria%20meningitidis%20%20SE%201-52-2019%20v2.pdf). Accessed November 18, 2021.

20. **New Zealand Ministry of Health. Invasive meningococcal disease report January-December 2019** (https://surv.esr.cri.nz/PDF_surveillance/MeningococcalDisease/2019/MeningococcalDisease_Q4_2019.pdf). Accessed July 14, 2022.

21. **Centers for Disease Control and Prevention. Enhanced Meningococcal Disease Surveillance Report, 2015** (https://www.cdc.gov/meningococcal/downloads/NCIRD-EMS-Report-2015.pdf). Accessed November 18, 2021.

22. **Federal Service for Supervision of Consumer Rights Protection and Human Welfare. State Report on the State of Sanitary and Epidemiological Well-Being of the Population in the Russian Federation in 2016-2018** (https://www.rospotrebnadzor.ru/upload/iblock/0b3/gosudarstvennyy-doklad-2016.pdf; https://www.rospotrebnadzor.ru/upload/iblock/d9d/gd_2017_seb.pdf; https://www.rospotrebnadzor.ru/upload/iblock/798/gosudarstvennyy-doklad-o-sostoyanii-sanitarno_epidemiologicheskogo-blagopoluchiya-naseleniya-v-rossiyskoy-federatsii-v-2018-godu.pdf). Accessed November 18, 2021.

23. **National Institute for Communicable Diseases. GERMS South Africa Annual Report 2010-2018** (https://www.nicd.ac.za/wp-content/uploads/2022/06/2010-GERMS-SA-Annual-report-Final.pdf; https://www.nicd.ac.za/assets/files/2011%20GERMS-SA%20Annual%20report%20pub%20final(1).pdf; https://www.nicd.ac.za/assets/files/2012%20GERMS-SA%20Annual%20Report.pdf; https://www.nicd.ac.za/assets/files/GERMS-SA%20AR%202013(1).pdf; https://www.nicd.ac.za/assets/files/GERMS-SA%20AR%202014(1)(1).pdf; https://www.nicd.ac.za/assets/files/2015%20GERMS-SA%20AR.pdf; https://www.nicd.ac.za/wp-content/uploads/2017/03/GERMS-SA-AR-2016-FINAL.pdf; https://www.nicd.ac.za/wp-content/uploads/2018/08/GERMS-SA-AR-2017-final.pdf; https://www.nicd.ac.za/wp-content/uploads/2019/11/GERMS-SA-AR-2018-Final.pdf). Accessed November 18, 2021.

24. **Laboratoire de Santé Publique de Québec. Bulletin STATLABO** (https://www.inspq.qc.ca/bulletin-statlabo/juillet-2011; https://www.inspq.qc.ca/bulletin-statlabo/juillet-2012; https://www.inspq.qc.ca/bulletin-statlabo/juillet-2013; https://www.inspq.qc.ca/bulletin-statlabo/juillet-2014; https://www.inspq.qc.ca/bulletin-statlabo/juillet-2015; https://www.inspq.qc.ca/bulletin-statlabo/juillet-2016; https://www.inspq.qc.ca/bulletin-statlabo/juillet-2017). Accessed November 17, 2021.

25. **Ministry of Health Israel. Jerusalem Central Laboratories - Annual Report 2010-2018** (https://www.health.gov.il/PublicationsFiles/LAB_JER2010.pdf; https://www.health.gov.il/PublicationsFiles/LAB_JER2011.pdf;

https://www.health.gov.il/PublicationsFiles/LAB_JER_2012.pdf;
https://www.health.gov.il/PublicationsFiles/LAB_JER2013.pdf;
https://www.health.gov.il/PublicationsFiles/LAB_JER2014.pdf;
https://www.health.gov.il/PublicationsFiles/LAB_JER2015.pdf;
https://www.health.gov.il/PublicationsFiles/LAB_JER2016.pdf;
https://www.health.gov.il/PublicationsFiles/LAB_JER2017.pdf;
https://www.health.gov.il/PublicationsFiles/LAB_JER2018.pdf). Accessed November 18, 2021.
